# The CTLH Ubiquitin Ligase Substrates ZMYND19 and MKLN1 Negatively Regulate mTORC1 at the Lysosomal Membrane

**DOI:** 10.21203/rs.3.rs-4259395/v1

**Published:** 2024-04-24

**Authors:** Yin Wang, Rui Guo, Brenda Iturbide Piedras, Hsin-Yao Tang, John M. Asara, Italo Tempera, Paul M. Lieberman, Benjamin E. Gewurz

**Affiliations:** Division of Infectious Diseases, Department of Medicine, Brigham and Women’s Hospital, 181 Longwood Avenue, Boston, MA 02115, USA; Department of Microbiology, Harvard Medical School, Boston, MA 02115, USA; Broad Institute of Harvard and MIT, Cambridge, MA 02142, USA; Division of Infectious Diseases, Department of Medicine, Brigham and Women’s Hospital, 181 Longwood Avenue, Boston, MA 02115, USA; Department of Microbiology, Harvard Medical School, Boston, MA 02115, USA; Broad Institute of Harvard and MIT, Cambridge, MA 02142, USA; Division of Infectious Diseases, Department of Medicine, Brigham and Women’s Hospital, 181 Longwood Avenue, Boston, MA 02115, USA; Department of Microbiology, Harvard Medical School, Boston, MA 02115, USA; Broad Institute of Harvard and MIT, Cambridge, MA 02142, USA; The Wistar Institute, Philadelphia, PA, USA; Division of Signal Transduction, Beth Israel Deaconess Medical Center and Department of Medicine, Harvard Medical School, Boston, MA, USA; The Wistar Institute, Philadelphia, PA, USA; The Wistar Institute, Philadelphia, PA, USA; Division of Infectious Diseases, Department of Medicine, Brigham and Women’s Hospital, 181 Longwood Avenue, Boston, MA 02115, USA; Department of Microbiology, Harvard Medical School, Boston, MA 02115, USA; Broad Institute of Harvard and MIT, Cambridge, MA 02142, USA; Program in Virology, Harvard Medical School

## Abstract

Most Epstein–Barr virus-associated gastric carcinoma (EBVaGC) harbor non-silent mutations that activate phosphoinositide 3 kinase (PI3K) to drive downstream metabolic signaling. To gain insights into PI3K/mTOR pathway dysregulation in this context, we performed a human genome-wide CRISPR/Cas9 screen for hits that synergistically blocked EBVaGC proliferation together with the PI3K antagonist alpelisib. Multiple subunits of carboxy terminal to LisH (CTLH) E3 ligase, including the catalytic MAEA subunit, were among top screen hits. CTLH negatively regulates gluconeogenesis in yeast, but not in higher organisms. Instead, we identified that the CTLH substrates MKLN1 and ZMYND19, which highly accumulated upon MAEA knockout, associated with one another and with lysosomes to inhibit mTORC1. ZMYND19/MKLN1 bound Raptor and RagA/C, but rather than perturbing mTORC1 lysosomal recruitment, instead blocked a late stage of its activation, independently of the tuberous sclerosis complex. Thus, CTLH enables cells to rapidly tune mTORC1 activity at the lysosomal membrane via the ubiquitin/proteasome pathway.

## Introduction

Gastric carcinoma is the fourth leading cause of cancer associated death worldwide^1^. The Cancer Genome Atlas defined four distinct gastric carcinoma subtypes, of which Epstein–Barr virus-associated gastric carcinoma (EBVaGC) represents ~9% of cases. Several features distinguish EBVaGC from the other subtypes, including particularly high frequency of non-silent mutations in *PIK3CA*, which encodes the phosphoinositide 3-kinase (PI3K) p110α catalytic subunit. As compared with 3–42% mutation frequency observed in other gastric carcinoma subtypes, *PIK3CA* mutations are present in >80% of EBVaGC, suggesting important oncogenic driver roles^2, 3^. PI3K negative regulator PTEN mutations are observed in other cases, raising the question of whether PI3K activation is an universal EBVaGC feature. Although curable if caught early, metastatic EBVaGC remains largely untreatable.

PI3K transduces signals from plasma membrane receptors to activate downstream anabolic metabolism pathways^4^. PI3K phosphorylates phosphatidylinositol 4,5-bisphosphate to generate the second messenger PIP3. Aberrant PI3K signaling is a cancer hallmark^5^. Major PI3K pathway targets include the kinases Akt and the mechanistic target of rapamycin complex 1 (mTORC1), comprised of mTOR, regulatory-associated protein of mTOR (Raptor), mammalian lethal with SEC13 protein 8 (mLST8), 40kDa Proline-rich Akt substrate (PRAS40) and DEP domain-containing mTOR-interacting protein (DEPTOR) subunits. mTORC1 is recruited to lysosomal outer membrane activation sites by Ras-related GTPase heterodimers RagA/C or RagB/D in response to nutrient cues, including plentiful amino acid supply^6, 7^. mTORC1 is then activated by the small GTPase Ras homolog enriched in brain (Rheb), which is tethered to lysosomal outer membrane sites^8–11^.

The PI3K-AKT-mTOR pathway plays a critical role in cell proliferation, survival and metabolism^12, 13^. Activated mTORC1 promotes anabolic metabolic pathways, including protein synthesis via phosphorylation of translation initiation factor 4E binding protein 1 (4EBP1) and S6 kinase (S6K), as well as nucleotide synthesis via upregulation of the transcription factor ATF4^14^. 4EBP1 phosphorylation upregulates cap-dependent translation, whereas S6K phosphorylation promotes protein synthesis and anabolic metabolism^15, 16^. Consequently, hyperactive PI3K activity is a major cancer therapeutic target^17^. However, drug resistance and dose-related toxicities limit the use of PI3K inhibitors^18^, which has stimulated enthusiasm for synergistic therapeutic approaches^19, 20^. For example, the Food and Drug Administration approved combination PIK3 p110α inhibitor alpelisib and estrogen receptor antagonist fulvestrant use for metastatic breast cancer^21^.

To gain insights into factors that support EBVaGC PI3K/mTORC1 signaling, we performed a human genome wide CRISPR/Cas9 screen for targets whose inhibition was synthetic lethal with alpelisib. Multiple subunits of the C-terminal to LisH (CTLH) E3 ligase were amongst the top screen hits. While CTLH negatively regulates gluconeogenesis in yeast^22–24^, this metabolism role has not been conserved in higher organisms, and potential CTLH roles in gastric carcinoma are unstudied. Our multi-omic analyses highlighted that CTLH supports mTORC1 activity by controlling levels of substrates ZMYND19 and MKLN1. Upon perturbation of CTLH activity, ZMYND19/MKLN1 associate at lysosome membrane sites, where they bind to Raptor and RagA/C to negatively regulate a late step of mTORC1 activation.

## Results

### CRISPR/Cas9 screen for alpelisib synthetic lethal targets

**Figure 1:**
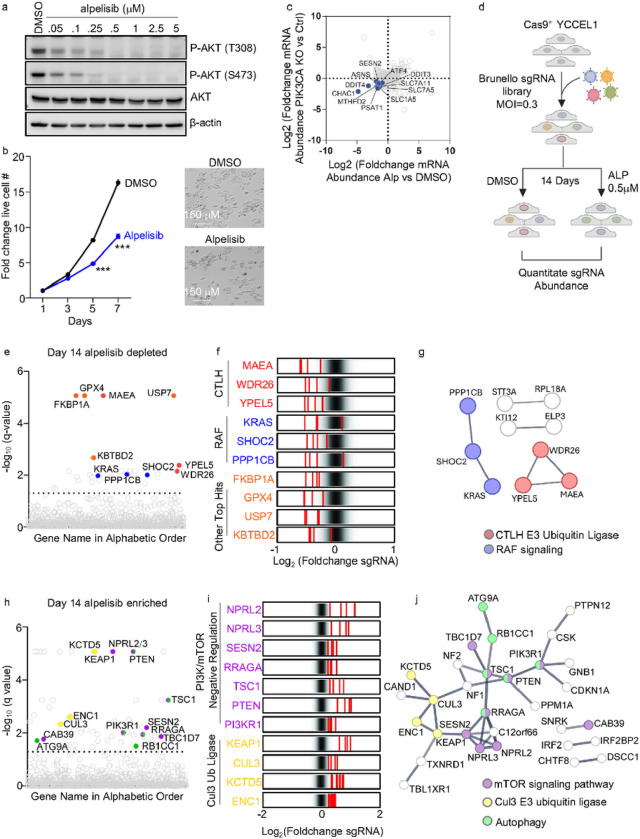
EBVaGC human genome-wide CRISPR-Cas9 screen for knockouts synthetic lethal with PI3K antagonist alpelisib. (A) Immunoblot analysis of alpelisib inhibition of PI3K substrate AKT phosphorylation, using whole cell lysates (WCL) from YCCEL1 treated with 0–5mM alpelisib for 3 hours. (B) Mean ± standard deviation (SD) fold change of live cell numberers of YCCEL1 cells treated with 0.5mM alpelisib vs DMSO vehicle from n=3 independent replicates. Representative bright-field images of YCCEL1 treated with DMSO or 0.5mM alpelisib for 7 days are also shown. (C) Volcano plot visualization of Log_2_ (fold change) RNA abundances from n=3 RNA-seq replicates of YCCEL1 CRISPR *PIK3CA* knockout (encodes the PI3K catalytic subunit) versus control cells (y-axis) and from cells treated with 5 mM alpelisib versus DMSO for 6 hours (x-axis). Values for individual transcripts are shown as circles. Values for the PI3K/mTOR pathway target transcription factor ATF4, and for selected ATF4 target genes, are highlighted in blue. (D) YCCEL1 human genome-wide CRISPR-Cas9 screen schematic diagram. Cas9+ YCCEL1 were transduced with the Brunello sgRNA library. Transduced cells were puromycin selected. At Day 7 post-transduction, cells were cultured in DMSO vehicle versus 0.5mM for 14 days. PCR-amplified sgRNA sequences were quantitated by next-generation DNA sequencing to identify hits, whose abundances were significantly higher in either group. The screen was performed in biological quadruplicate. (E) CRISPR screen Manhattan plot demonstrating −Log_10_ multiple hypothesis test adjusted q-values, as defined by the STARs algorithm. Genes are arranged by alphabetical order along the x-axis. Each circle depicts values for a human gene target. Lower values signify depletion of sgRNAs targeting the indicated gene in Day 14 alpelisib versus DMSO treated cells. Screen hits encoding CTLH subunits are colored red, hits encoding RAF-related signaling are colored blue, and other selected top hits are colored orange. (F) Rug plots showing the Log2 transformed Foldchange abundances in Day 14 alpelisib versus DMSO treated cells of the four sgRNAs targeting the indicated screen hit gene (shown in red), relative to the overall distribution of Brunello library sgRNAs. CTLH subunits, RAF-related and other top screen hits are highlighted. (G) STRING network analysis showing selected high confidence score (>0.75) connections between selected screen hits. (H) CRISPR screen Manhattan plot demonstrating −Log_10_ multiple hypothesis test adjusted q-values, as defined by the STARs algorithm. Genes are arranged by alphabetical order along the x-axis. Each circle depicts values for a human gene target. higher values signify enrichment of sgRNAs targeting the indicated gene in Day 14 alpelisib versus DMSO treated cells. Screen hits encoding PI3K/mTOR negative regulators are colored purple, hits encoding Cul3 ubiquitin ligase related proteins are in colored yellow, and autophagy related genes are in shown in green. (I) Rug plots showing the Log2 transformed Foldchange abundances in Day 14 alpelisib versus DMSO treated cells of the four sgRNAs targeting the indicated screen hit gene (shown in red), relative to the overall distribution of Brunello library sgRNAs. PI3K/mTOR negative regulators and Cul3-related screen hits are highlighted. (J) STRING network analysis showing selected high confidence score (>0.75) connections between selected screen hits.

We characterized PI3K signaling in YCCEL1, one of only three available EBVaGC cell lines, which expresses the EBV-encoded proteins EBNA1 and LMP2A^25^ and harbors a PIK3CA kinase domain histidine 1047 arginine gain-of-function mutation (**Extended Data Fig. S1a**). Alpelisib diminished PI3K target AKT threonine 308 and serine 473 phosphorylation in a dose-dependent manner, with most AKT phosphorylation lost at the 0.5mM dose in YCCEL1 (Fig. 1a). At this dose, alpelisib restrained YCCEL1 proliferation by approximately 50% (Fig. 1b). Alpelisib treatment and CRISPR *PIK3CA* knockout (KO) produced concordant transcriptome-wide changes in YCCEL1, suggestive of on-target activity (Fig. 1c, **Extended Data Fig. S1b-c, Extended Data Table S1**).

To identify EBVaGC targets that synergistically block EBVaGC proliferation together with 0.5 mM alpelisib, we performed a human genome-wide CRISPR-Cas9 screen. Cas9+ YCCEL1 were transduced with the Brunello lentiviral single guide RNA (sgRNA) library at multiplicity of infection of 0.3. 7 days post-transduction and following puromycin selection, the KO library was cultured either with 0.5mM alpelisib or with DMSO vehicle for an additional two weeks. Using biological triplicate replicates, PCR-amplified sgRNA abundances in the surviving cell pools were quantitated by next-generation DNA sequencing. sgRNA abundances between alpelisib versus DMSO treated cells were cross-compared, and hits were identified by the STARS algorithm (Fig. 1d, **Extended Data Table S2**)^26^.

At a multiple hypothesis adjusted q<0.05 cutoff, the screen identified 30 hits, in which independent sgRNAs targeting these human genes were depleted in the surviving alpelisib-treated cell pool relative to their levels in DMSO treated cells (Fig. 1e). Top hits included genes encoding factors known to be highly related to PI3K biology. These included 1) the ubiquitin E3 ligase KBTBD2, which regulates PI3K p85α regulatory subunit abundance^27, 28^; 2) GPX4, which uses glutathione to detoxify lipid free radicals and protect against ferroptosis^29^; and 3) the ubiquitin specific protease USP7, which removes a monoubiquitin group from PTEN to support its cytoplasmic subcellular localization^30^ and which protects gastric cancer cells from ferroptosis^31^ (Fig. 1e–f). These data are consistent with the observation that PI3K signaling protects cells against ferroptosis induction^34^.

Multiple positive regulators of the RAF/MAP kinase pathway also scored strongly, including KRAS, which recruits RAF to plasma membrane activation sties, as well as the catalytic and regulatory components of the SHOC2 phosphatase complex, encoded by *SHOC2* and *PPP1CB* (Fig. 1e–g). SHOC2 dephosphorylates plasma membrane localized RAF phosphoserine 365 to drive RAF/MAP kinase pathway activation^32^. While PI3K/mTOR and RAS/MAPK pathways crosstalk^33, 34^, this has yet to be characterized in the gastric carcinoma setting. However, KRAS is targeted by deleterious mutations in multiple gastric carcinoma subtypes including EBVaGC^2^. We validated that GPX4, USP7 or KRAS KO significantly reduced numbers of live YCCE1L treated with alpelisib, relative to levels in cells treated with vehicle. We also validated that the ferroptosis antagonist Fer-1 rescued survival of cells treated with both alpelisib and with the GPX4 antagonist ML-210 (**extended data Fig. 1d–f**).

Multiple subunits of the E3 ubiquitin ligase C-terminal to LisH (CTLH) complex scored as top hits, including genes encoding the catalytic unit MAEA subunit, YPEL5 and WDR26 (Fig. 1 e–g). The CTLH E2 ubiquitin ligase encoded by *UBE2H* nearly also scored. CTLH is the mammalian homologue of the *Saccharomyces cerevisiae* glucose-induced degradation deficient (GID) complex, which ubiquitinates gluconeogenic enzymes when extracellular glucose is abundant^22, 35–37^. This metabolic role does not appear to have been conserved in higher organism CTLH complexes.

The CRISPR screen also identified sgRNA targets whose knockout ameliorated effects of low dose alpelisib on YCCEL1 proliferation, as judged by significantly enriched sgRNA abundance in alpelisib versus DMSO treated cells (Fig. 1h–j, **Extended Data Table S2**). Top hits included genes encoding the PI3K negative regulator PTEN and the PI3K p85a regulatory subunit (*PIK3R1*), as expected. p85a restrains PI3K catalytic activity and is a tumor suppressor^38, 39^. Multiple mTOR negative regulators were also amongst top hits in this category, including TSC1 and components of the GATOR1 complex encoded by NPRL2, NPRL3 and SESN2. Together with TSC2 and TBC1D7, TSC1 negatively regulates mTORC1 activation through GTPase-activating protein (GAP) activity towards the small GTPase Rheb^40^, whereas GATOR1 GTPase-activating protein activity negatively regulates mTORC1 lysosomal recruitment by the regulator/Rag complex in response to amino acid depletion^8, 41–43^. Multiple Cullin-3 (Cul-3) E3 ligase related genes also scored (Fig. 1h–j). The AKT pathway negative regulator KCTD5, which is a Cul3 substrate adaptor, scored strongly^44^. Similarly, the Cul3 substrate adaptor KEAP1 and the kelch-family member ENC1 scored, a target of which is the transcription factor Nrf2, which drives antioxidant responses^45^.

### CTLH inhibition causes widespread metabolic remodeling that is exacerbated by alpelisib

**Figure 2:**
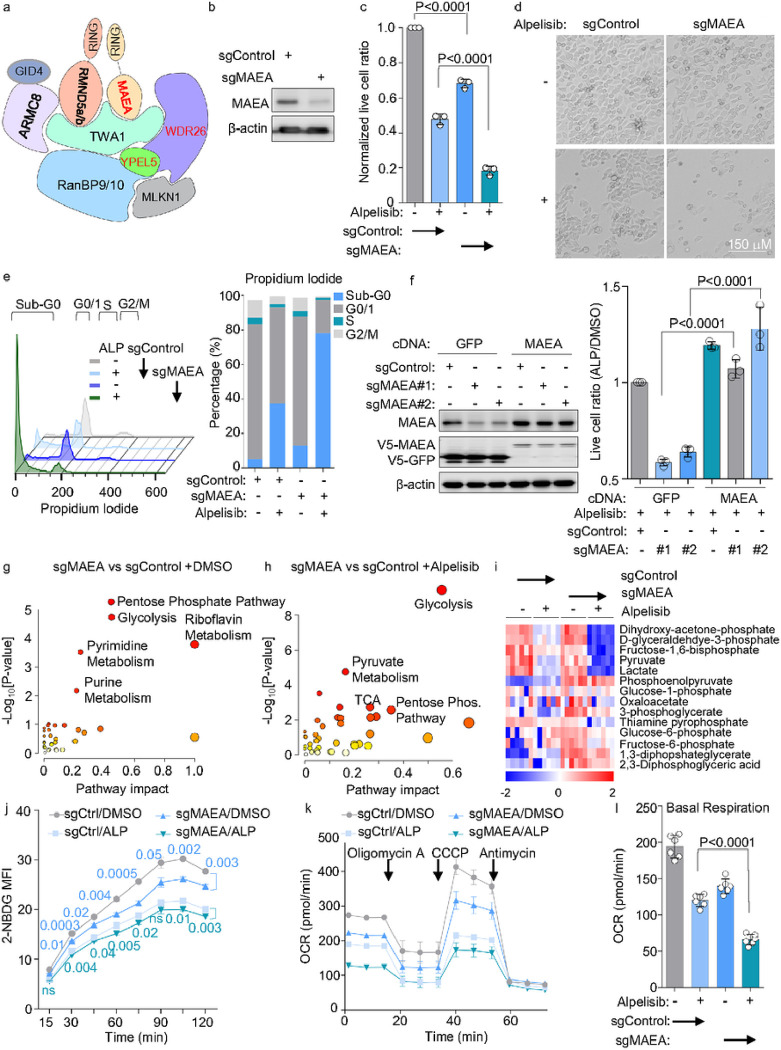
Key CTLH E3 ubiquitin ligase roles in EBVaGC survival and metabolism regulation. (A) Schematic diagram of CTLH E3 ubiquitin ligase, adapted from Sherpa *et al* ^82^. Screen hits are shown in red. (B) Immunoblot of WCL from YCCEL1 expressing control vs. MAEA sgRNAs for 7 days. (C) Normalized mean ± SD live cell ratios from n=3 replicates of YCCEL1 expressing control or MAEA sgRNA and treated with 0.5mM alpelisib for 7 days, as indicated. (D) Representative bright-field images of YCCEL1 expressing control or MAEA sgRNAs and treated with DMSO vehicle or 0.5mM alpelisib for 7 days. (E) Cell cycle analysis of propidium iodide (PI) stained YCCEL1 treated with 0.5mM alpelisib and expressing sgRNAs for 7 days, as indicated. Show to the right are mean percentages from n=3 replicates of cells in sub-G0, G0/1, S and G2/M cell cycle phases, based on FACS analyses as show to the left. (F) MAEA cDNA rescue. Left, immunoblot analysis of WCL from YCCEL1 expressing control GFP vs MAEA rescue cDNA with PAM site point mutation to abrogate CRISPR editing, together with control or either of two independent screen hit MAEA sgRNAs for 7 days. Right, Mean ± SD live cell ratios from n=3 replicates of YCCEL1 expressing control GFP vs. MAEA rescue CDNA and treated with 0.5mM alpelisib vs. DMSO for 7 days. Numbers were normalized to levels in cells expressing GFP and control sgRNA. (G) Metabolic pathway impact analysis of YCCEL1 MAEA depletion. Shown is the metabolic pathway impact map from liquid chromatography mass spectrometry (LC/MS) analyses (n=6 independent replicates) of YCCEL1 expressing MAEA vs control sgRNAs for 7 days in the presence of DMSO vehicle. x axis shows pathway impact values, y axis shows the −log_10_ P-value from MetaboAnalyst 3.0^83^ topologic analysis of the metabolomic datasets. (H) Metabolic pathway impact analysis of YCCEL1 MAEA depletion together with alpelisib. Shown is the metabolic pathway impact map from LC/MS analyses (n=6 independent replicates) of YCCEL1 expressing MAEA vs control sgRNAs for 7 days, grown in the presence of 0.5 mM alpelisib for 30 hours. (I) Heatmap analyses of glycolysis pathway metabolite row Z-scores from LC/MS analysis of YCCEL1 cells expressing MAEA versus control sgRNAs and grown in the presence of 0.5 mM alpelisib, as indicated. Z-scores show standard deviation(s) of metabolite abundances from the mean value in each row. (J) MAEA KO and alpelisib effects on glucose uptake. FACS analysis of 2-NBDG uptake (as a measure of glucose uptake) in YCCEL1 expressing either Control (Ctrl) or MAEA sgRNAs and treated with DMSO or alpelisib (ALP). Shown means ± SD mean fluorescence intensity (MFI) values from n=3 independent replicates. (K) Seahorse analysis of oxygen consumption rates (OCRs) of YCCEL1 as in (J), in the presence ATP synthase in inhibitor oligomycin A, uncoupling agent CCCP or electron transport chain complex III inhibitor antimycin. Shown are the mean ± SEM from n=6 independent replicates. (L) Basal OCR of YCCEL1 expressing control or MAEA sgRNAs and treated with alpelisib for 7 days, as indicated. Shown are mean ± SD values from n=6 independent replicates. Blots are representative of n=3 replicates. P-values were calculated by Student’s t test.

CTLH is an unusually complex ubiquitin E3 ligase, comprised of at least nine components (Fig. 2a), but has not previously been linked to PI3K signaling or studied mechanistically in the gastric cancer context. We therefore investigated phenotypes of CRISPR KO of the RING domain-containing catalytic subunit and screen hit MAEA. MAEA KO and alpelisib together decreased YCCEL1 live cell numbers more than either alone (Fig. 2b–d). Similar results were obtained in EBV-uninfected *PIK3CA* mutant HGC-27 gastric carcinoma and in *PIK3CA* wildtype SNU-1 gastric carcinoma cells (**Extended data Fig. S2b-c**). Notably, SNU-1 harbor an activating *KRAS* G12D mutation that activates downstream PI3K and MAPK pathways^46^. However, alpelisib and MAEA KO exerted milder effects on EBV-uninfected SNU-16 gastric carcinoma cells, perhaps due to lower levels of PI3K activity (**Extended data Fig. S2d**).

To identify how MAEA KO altered gastric carcinoma growth versus survival, alone or together with alpelisib, we performed cell cycle analysis on propidium iodide stained cells. While MAEA KO or alpelisib alone diminished S-phase cell number, together they caused widespread cell death, as judged by the number of sub-G0 cells (Fig. 2e). CRISPR MAEA effects were on-target, since MAEA cDNA with a silent point mutation to abrogate Cas9 cutting rescued survival of alpelisib-treated YCCE1L upon KO of endogenous MAEA (Fig. 2f).

Given ancestral and known CTLH metabolic roles^47–49^, we hypothesized that CTLH perturbation altered metabolism pathways, and that this underlay its strong screen phenotype. To gain insights, we performed targeted liquid chromatography-tandem mass spectrometry (LC-MS/MS) profiling^50^ of control vs MAEA depleted cells early after CRISPR editing, in the absence or presence of 0.5mM alpelisib for 30 hours. MAEA editing most significantly perturbed glycolysis, pentose phosphate, riboflavin, pyrimidine and purine metabolism pathways (Fig. 2g, **Extended Data Table S3**). Glycolysis and pyruvate metabolism where the most significantly altered metabolism pathways in alpelisib-treated MAEA KO vs control cells (Fig. 2h, **Extended Data Table S3**). Nucleotide metabolism and glycolysis were also the most significantly perturbed by alpelisib in both MAEA KO or control YCCEL1 cells (**Extended data Fig. S2e-f, Extended Data Table S3**), further highlighting overlap in effects of MAEA perturbation and PI3K blockade.

Multiple glycolysis pathway metabolites were highly depleted by MAEA KO + alpelisib treatment, including dihydroxy-acetone-phosphate, D-glyceraldehyde-3-phosphate, fructose-1,6-biphosphate, pyruvate and lactate (Fig. 2i, **Extended Data Table S3**). Similarly strong effects were observed on purine and pyrimidine pathway metabolites (**Extended data Fig. S2g**). To further analyze effects of MAEA KO on glycolysis, alone or together with alpelisib, we performed FACS analysis of fluorescent glucose analog 2-NBDG uptake. MAEA KO diminished 2-NBDG levels, alone or in combination with alpelisib (Fig. 2j). Furthermore, Seahorse XF flux analysis demonstrated that MAEA editing reduced extracellular acidification, basal and maximum respiration and ATP production, either alone or additively with alpelisib (**Fig. j-l, Extended data Fig. 2h-k**). Taken together, these data suggest that CTLH supports major central carbon and anabolic metabolism pathways.

### CTLH substrates ZMYND19 and MKLN1 form a complex that negatively regulates mTOR

**Figure 3:**
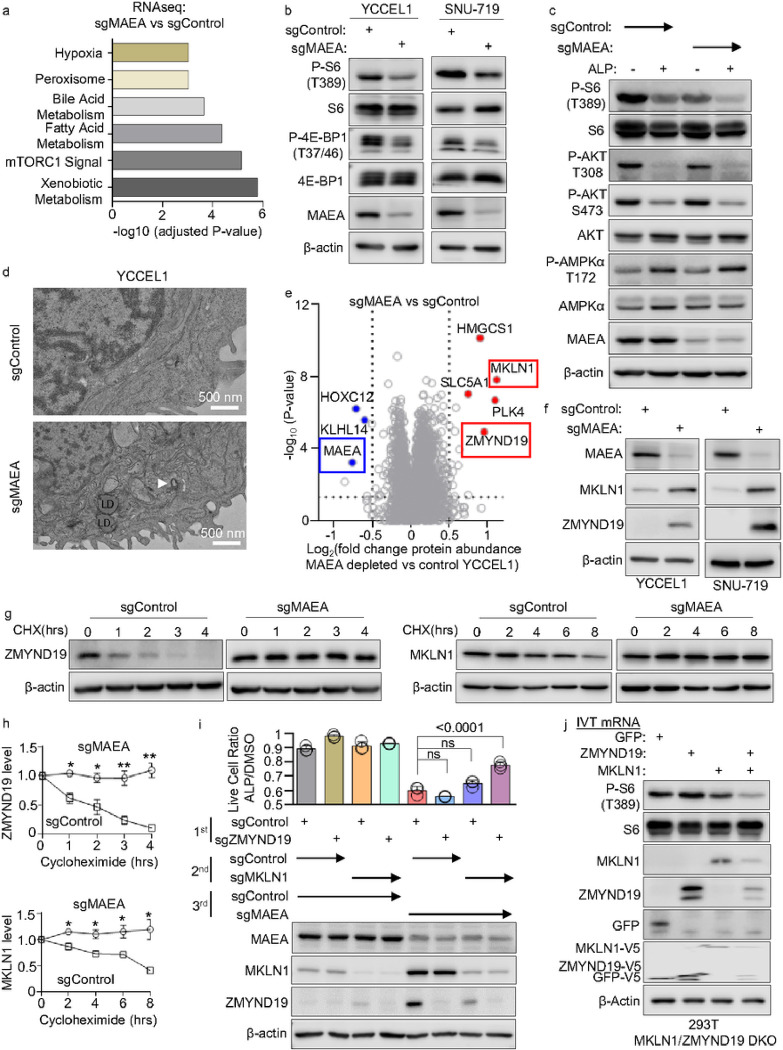
CTLH substrates ZMYND19 and MKLN1 inhibit mTOR. (A) MSigDB hallmark pathway analysis of differentially expressed genes (log_2_ foldchange mRNA abundance >0.5 or < −0.5 and with adjusted P-value < 0.05) between YCCEL1 expressing MAEA vs control sgRNAs for 7 days. Gene set enrichment analysis was performed with Enrichr^81^. (B) Immunoblot analysis of WCL from EBV+/Cas9+ YCCEL1 or SNU-719 gastric carcinoma cells expressing control or MAEA sgRNAs for 5 days. (C) Immunoblot analysis of WCL from YCCEL1 expressing control or MAEA sgRNA for 7 days and treated with alpelisib 0.5mM for 8 hours, as indicated. (D) Transmission electron micrographs of YCCEL1 expressing control vs MAEA sgRNAs for 7 days. Representative images from >10 fields from n=2 replicates are shown. Scale bar, 500 nm. LD, lipid droplet. White arrowhead indicates a double membrane structure. (E) Volcano plot of whole cell proteomic analysis of MAEA depleted vs control cells to identify candidate YCCEL1 substrates. Shown are − log10 (P-value) y-axis vs Log2 (fold change in protein abundance) x-axis between YCCEL1 that expressed MAEA vs control sgRNA for 7 days, from n=3 independent replicates. MAEA was amongst the most depleted protein by CRISPR editing, as expected. ZMYND19 and MKLN1 were amongst the most significantly upregulated by MAEA depletion. (F) Immunoblot analysis of WCL from YCCEL1 (left) or SNU-719 (right) expressing control or MAEA sgRNA for 7 days and treated with cycloheximide for the indicated number of hours. (G) Validation of proteomic data. Immunoblot analysis of WCL from YCCEL1 expressing control vs MAEA sgRNAs for 7 days. (H) Mean ± SD ZMYND19 (upper) or MKLN1 (lower) abundances in YCCEL1 expressing MAEA vs control sgRNAs. Shown are b-actin normalized abundances from n=3 immunoblots, analyzed with Licor Image Studio software. (I) Live cell ratios of YCCEL1 expressing the indicated control, ZMYND19 and/or MKLN1 sgRNAs and then treated with 0.5 mM alpelisib vs DMSO for 5 days. Shown are mean ± SD values from n=3 independent replicates of alpelisib vs DMSO treated cells. Bottom, immunoblot analysis of WCL from cells expressing the indicated combinations of three sgRNAs, which were sequentially expressed by lentiviral transduction, just prior to alpelisib vs DMSO treatment. (J) mTOR pathway signaling in 293T cells double knocked out (DKO) of endogenous MKLN1 and ZMYND19, which then overexpressed MKLN1 or ZMYND19 with in vitro transcription (IVT) system. In vitro transcribed mRNAs of GFP, ZMYND19, MKLN1, and ZMYND19/MKLN1 were transfected into the DKO cells with Neon electroporation transfection. The cells were collected after 3 hours of transfection. β-Actin was used as the loading control. Blots are representative of n=3 independent replicates. P-values were calculated by Student’s t-test, ns=non-significant.

To gain further insights, we next performed RNAseq on control vs MAEA KO YCCEL1. mTORC1 and xenobiotic metabolism pathways were the most significantly altered by MAEA depletion (Fig. 3a, **Extended Data Table S4**). Multiple ATF4 regulated genes were highly downmodulated in alpelisib treated MAEA KO cells, even in comparison with alpelisib-treated control cell levels (**Extended data Fig. S3a, Extended Data Table S4**). mTORC1 can induce purine synthesis through one-carbon metabolism via upregulation of methylenetetrahydrofolate dehydrogenase 2 (MTHFD2) in an ATF4-dependent manner^51, 52^. Since ATF4 and MTHFD2 were highly downmodulated in alpelisib-treated MAEA KO cells (**Extended data Fig. S3a, Extended Data Table S4**), we hypothesized that they may jointly block mTOR. Consistent with this, MAEA KO diminished translation rate, as judged by puromycin chase analysis (**Extended data Fig. S3b**). By contrast, MAEA KO, with or without alpelisib, did not increase EIF2a serine 51 phosphorylation, suggesting that an integrated stress response pathway was not responsible for effects on translation (**Extended data Fig. S3c**).

We therefore next analyzed MAEA KO effects on mTOR activity. MAEA KO reduced phosphorylation of mTOR target S6 kinase and 4E-BP1 phosphorylation (Fig. 3b). Further suggestive of a CTLH regulatory role in support of mTORC1, MAEA depletion and alpelisib additively decreased S6 T389 phosphorylation. Although the GID complex regulates AMP kinase in *C. elegans*^49^, we did not appreciate a significant change in AMPK T172 phosphorylation in MAEA depleted cells (Fig. 3c, **Extended data Fig S3d**). Also suggestive of inhibitory effects at the level of mTORC1, MAEA KO and low dose rapamycin additively reduced S6 phosphorylation levels and live cell numbers (**Extended data Fig. S3e-f**). Furthermore, transmission electron microscopy demonstrated lipid droplets and double membrane structures in MAEA depleted cells, consistent with decreased mTOR activity and increased autophagy (Fig. 3b).

To screen for CTLH substrates that regulate mTOR activity, we performed whole cell proteomic analysis. Indicative of on-target CRISPR editing, MAEA itself was one of the most significantly depleted proteins in cells expressing MAEA sgRNA relative to control cells. Consistent with prior studies^53, 54^, the abundances of MKLN1 and ZMYND19 were amongst the most significantly increased by CTLH perturbation (Fig. 3e, **Extended Data Table S5**), which we validated by immunoblot in both YCCEL1 and SNU-719 EBVaGC cells (Fig. 3f). Likewise, ZMYND19, and to a somewhat lesser extent MKLN1 half-lives were 1.9 and 7.7 hours, respectively in control cells, but each were significantly stabilized by MAEA KO (Fig. 3g–h). Bortezomib proteasome inhibition increased steady state MKLN1 and ZMYND19 levels, and also increased levels of inducibly expressed ZMYND19 or MKLN1, whose steady state levels were otherwise low (**Extended Data Fig. S3g-i**). Interestingly, MKLN1 is a component of the CTLH complex, and MKLN1 depletion also increased steady-state ZMYND19 levels, suggesting that it supports ZMYND19 turnover (**Extended data Fig. S3j**). MAEA KO also significantly increased MKLN1 and ZMYND19 levels in alpelisib treated cells. By contrast, alpelisib alone did not increase MKLN1 or ZMYND19 abundances, whereas alpelisib treatment did not further increase ZMYND19 or MKLN1 levels in MAEA depleted cells (**extended data Fig. S3k-m, Extended Data Table S5**).

We next tested whether ZMYND19 and/or MKLN1 were necessary for YCCEL1 cell death triggered by MAEA KO plus alpelisib. We expressed control sgRNA or sgRNA targeting ZMYND19, MKLN1 or MAEA and then treated edited cells with alpelisib or DMSO for 7 days. As shown in Fig. 3i, while KO of either ZMYND19 or MKLN1 alone failed to rescue alpelisib-treated MAEA KO cell survival, ZMYND19/MKLN1 double KO significantly rescued alpelisib-treated live cell numbers, suggesting a partially redundant or joint role (Fig. 3i). Furthermore, electroporation of *in vitro* transcribed (IVT) ZMYND19 mRNA (used to overcome its proteasomal turnover) decreased mTOR target S6 T389 phosphorylation levels, whereas combined ZMYND19 and MKLN1 mRNA electroporation decreased S6 T389 phosphorylation (Fig. 3j, **Extended data Fig. S3n**). Collectively, these data suggest that ZMYND19 and MKLN1 together inhibit mTOR.

### CTLH, ZMYND19 and MKLN1 subpopulations localize to lysosomal membranes

**Figure 4:**
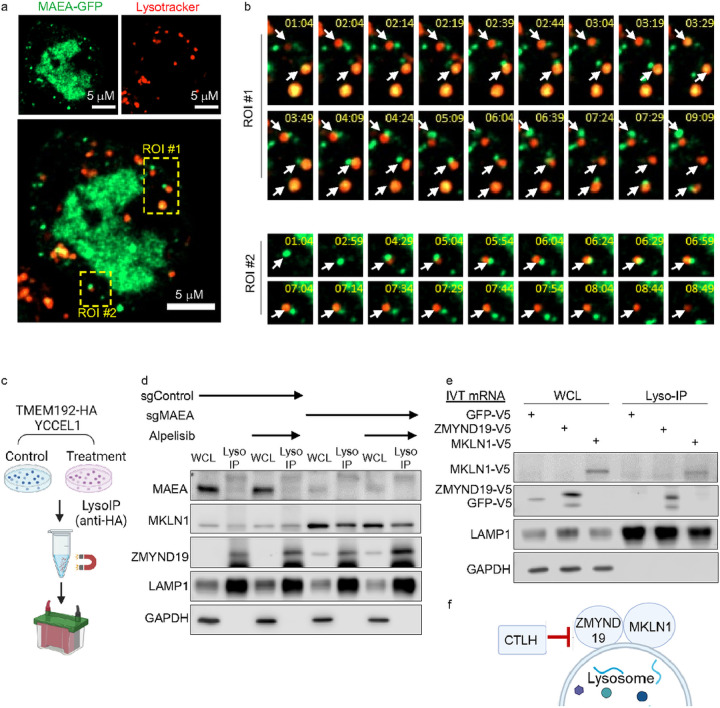
CTLH substrates ZMYND19 and MKLN1 associate with lysosomes upon CTLH inhibition. (A) Confocal microscopy analysis of CTLH subcellular distribution. YCCEL1 expressing GFP-tagged MAEA for 24 hours were stained with LysoTracker red. (B) Two regions of interest (ROI) marked in panel A were analyzed over an ~8 minute timecourse to analyze for dynamic association between MAEA-GFP and lysosomes. (C) Schematic diagram of lysosomal immunopurification approach (LysoIP)^57^. HA-epitope tagged lysosomal transmembrane protein TMEM192 was stably expressed in YCCEL1. TMEM192HA decorated lysosomes were immunopurified with anti-HA magnetic beads. (D) Immunoblots of WCL vs LysoIP samples from YCCEL1 TMEM192-HA expressing cells that also expressed control or MAEA sgRNAs and that were treated with 0.5 mM alpelisib for 24 hours, as indicated. GAPDH and LAMP1 were used as fractional controls for cytosolic vs lysosomal proteins, respectively. (E) Immunoblots of WCL vs LysoIP samples from YCCEL1 transfected with in vitro transcribed (IVT) mRNAs encoding V5-tagged GFP, ZMYND19 or MKLN1. (F) Schematic model. A cytosolic subpopulation of CTLH targets lysosome-associated ZMYND19 and MKLN1 for proteasomal degradation.

Lysosomal localization controls mTORC1 activation. We therefore investigated CTLH subcellular distribution, using GFP-tagged MAEA as a readout in live YCCEL1. Consistent with previous studies^55, 56^, the majority of MAEA localized to the nucleus, but cytoplasmic subpopulations were also observed (Fig. 4a). To better characterize the cytoplasmic puncta, we stained lysosomes with LysoTracker Red in cells expressing MAEA-GFP. Interestingly, a subset of MAEA-GFP puncta dynamically abutted lysosomes (Fig. 4a–b, **Extended data Fig. S4a, Extended Data Movie**), suggesting that CTLH may be transiently associate with a lysosomal outer membrane region substrate.

To further test the hypothesis that ZMYND19 and/or MKLN1 localize to lysosomal outer membrane regions, we used lysosomal immunopurification^57^. We established YCCEL1 with stable expression of integral lysosomal transmembrane protein 192 (TMEM192) tagged with three tandem HA-epitopes, which were exposed to the cytosol (HA-Lyso cells)^57^ (Fig. 4c). Immunoblot analysis of whole cell lysate (WCL) versus lysosome immunoprecipitation (Lyso-IP) samples suggested appropriate fractionation, as lysosomal-associated membrane protein 1 (LAMP1) was enriched in Lyso-IP samples as compared with whole cell lysates, whereas GAPDH was not detected by immunoblot of Lyso-IP samples (Fig. 4c–d). We detected MAEA in WCL, but not appreciably in the Lyso-IP fraction. However, MKLN1 was readily detectable in both WCL and Lyso-IP samples of MAEA KO cells, whereas ZMYND19 was preferentially detected in the Lyso-IP fraction (Fig. 4d). Alpelisib did not appreciably alter MKLN1 or ZMYND19 association with lysosomes. Similar results were obtained in 293T cells (**Extended Data Fig. S4b**). Transiently expressed ZMYND19 and MKLN1 were highly enriched in Lyso-IP samples, whereas negative control GFP was not (Fig. 4e). Taken together, these data suggest that ZMYND19 recruits MKLN1 to lysosomal outer membrane regions (Fig. 4f).

### ZMYND19 and CTLH Domains Important for Association and mTOR blockade

**Figure 5:**
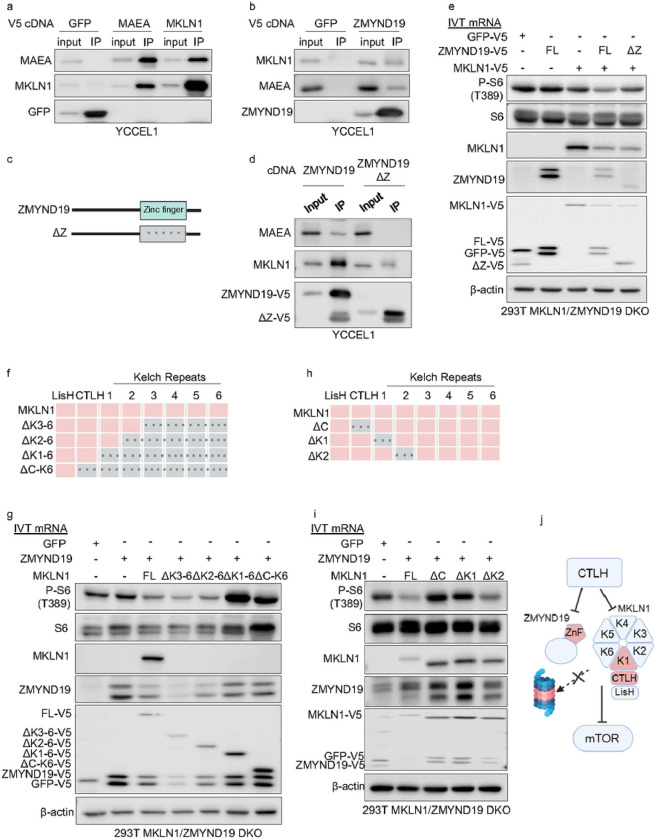
Identification of ZMYND19 and MKLN1 domains important for mTOR inhibition. (A) MAEA and MKLN1 co-immunoprecipitation analysis. Immunoblots of 5% input vs anti-V5 immunopurified V5-GFP, V5-MAEA or V5-MKLN1 from YCCEL1 that stably expressed the indicated cDNAs. (B) MAEA and ZMYND19 co-immunoprecipitation analysis. Immunoblots of 5% input vs anti-V5 immunopurified V5-GFP or V5-ZMYND9 from YCCEL1 that stably expressed the indicated cDNAs. (C) Schematic model of ZMYND19 full length and zinc finger domain deletion mutant ZMYND19 (ΔZ). Zinc finger deletion is indicated by gray box with dotted lines. (D) Analysis of ZMYND19 zinc finger role in association with MKLN1 and MAEA. Immunoblots of 5% input vs anti-V5 immunopurified full length versus ΔZ ZMYND19 from YCCEL1 that stably expressed the indicated cDNAs. (E) Analysis of ZMYND19 zinc finger role in mTOR inhibition. Immunoblot analysis of 293T MKLN1/ZMYND19 double knockout (DKO) single cell clines electroporated with the indicated *in vitro* transcribed (IVT) mRNAs encoding V5-tagged GFP, full length (FL) or ΔZ ZMYND19. WCL were prepared three hours after electroporation. (F) Model of full length MKLN1 and C-terminal truncation mutants, showing the LisH, CTLH and six kelch domains. Domain deletions are indicated by gray boxes with dotted lines. (G) Analysis of MKLN1 kelch and CTLH (C) domain roles in mTOR blockade. Immunoblot analysis of 293T MKLN1/ZMYND19 DKO clones electroporated with the indicate IVT mRNAs. WCL were prepared three hours after electroporation. (H) Model of full length MKLN1 and CTLH, Kelch domain 1 (ΔK1) or 2 (ΔK2) deletion mutants. (I) Analysis of mTOR blockade by MKLN1 deletion mutants lacking the CTLH (ΔC), kelch 1 (ΔK1) or kelch 2 (ΔK2) domains. Immunoblot analysis of 293T MKLN1/ZMYND19 DKO clones electroporated with the indicate IVT mRNAs. WCL were prepared three hours after electroporation. (J) Model of mTOR blockade by the ZMYND19/MKLN1 complex. CTLH negatively regulates ZMYND19 and MKLN1. The ZMYND19 zinc finger (ZnF) is important for association with MKLN1. The MKLN1 K1 and CTLH domains are important for mTOR inhibition. Blots are representative of n=3 replicates.

To gain further insights into physical association between MAEA, MKLN1 and ZMYND19, we used co-immunoprecipitation analysis. MAEA and MKLN1 reciprocally co-immunoprecipitated one another, but not with control GFP (Figure 5a). Similarly, MAEA and MKLN1 co-immunoprecipitated with V5-tagged ZMYND19 (Figure 5b). To test if the ZMYND19 C-terminal zinc finger was required for association with MKLN1 or MAEA, we expressed V5-tagged full length or zinc finger deleted ZMYND19 (DZnF, Fig. 5c) in YCCEL1. Zinc finger deletion reduced, but did not complete abrogate MKLN1 co-immunoprecipitation with ZMYND19, whereas it strongly perturbed association between ZMYND19 and MAEA in both YCCEL1 and 293T (Fig. 5d, **Extended data Fig. S5a**). However, DZnF ZMYND19 maintained the ability to associate with lysosomes, albeit perhaps somewhat less robustly (**Extended Data Fig. S5b**). These data suggests that the ZMYND19 zinc finger mediates association with both MAEA and MKLN1, the latter of which is a component of CTLH.

To then test ZYMND19 and/or MKLN1 effects on mTOR blockade, we electroporated a 293T single cell MKLN1/ZMYND19 double KO clone with control GFP, ZMYND19 and/or MKLN1 mRNAs. Co-expressed ZMYND19 and MKLN1 impaired mTOR activity, as judged by a reduction in S6 phosphorylation, whereas it was more mildly impaired by MKLN1 when expressed alone or in combination with DZnF ZMYND19 (Fig. 5e). ZMYND19 expressed alone did not appreciably block S6 phosphorylation (Fig. 5e).

We next examined MKLN1 domains important for mTOR blockade. MKLN1 is comprised of an N-terminal LisH domain, a CTLH domain and six Kelch repeats (Fig. 5f), the latter of which can assemble b-propeller structures to support protein–protein interactions^58^. We therefore tested the effects of progressive C-terminal deletion of MKLN1 kelch repeats on its ability to inhibit mTOR in combination with full length ZMYND19. This analysis indicated that MKLN1 kelch repeats 2–6 were dispensable for mTOR blockade, as judged by inhibition of S6 phosphorylation (Fig. 5g). Interestingly, Lyso IP demonstrated that Dkelch2–6 and in particular Dkelch1–6 increased the fraction of MKLN1 associated with lysosomes (**Extended data Fig. S5c**). However, expression of ZMYND19 together with MKLN1 C-terminal deletion mutants lacking all six Kelch repeats, or lacking the CTLH domain and the six kelch repeats, resulted in S6 hyperphosphorylation. Notably, ZMYND19 steady state levels were lower when co-expressed with MKLN1 constructs that that diminished mTOR activity (full length, Dkelch3–6 or Dkelch2–6) (Fig. 5g). These data indicate that the first MKLN1 kelch repeat is needed for mTOR inhibition, but not for lysosomal association.

We next tested whether the MKLN1 CTLH domain was necessary for mTOR blockade, and whether MKLN1 kelch repeat 1, or rather any MKLN1 kelch repeat, was necessary for mTOR blockade. To do so, we expressed *in vitro* transcribed RNAs encoding ZMYND19 together with full length MKLN1 or with MKLN1 deletion mutants lacking the CTLH domain, kelch repeat 1 or kelch repeat 2 in 293T MKLN1/ZMYND19 double KO cells. This analysis indicated that the CTLH domain was necessary for mTOR inhibition. Likewise, since kelch 1 but not kelch repeat 2 was required for mTOR inhibition, our results suggest that kelch repeat 1 rather is uniquely required (Fig. 5h–i). As described above, steady state ZMYND19 levels were higher when co-expressed with MKLN1 deletion mutants that fail to block mTOR (Fig. 5i, lanes 4–6). Interestingly however, each of these deletion mutants co-immunoprecipitated with ZMYND19-HA (**Extended data Fig. S5d**). Taken together, these results are consistent with a model in which MKLN1 kelch repeats 1–6 downmodulate its recruitment to lysosomes, but that association with the ZMYND19 zinc finger domain relieves this inhibition, and that the ZMYND19/MKLN1 complex blocks mTOR in a manner dependent on the MKLN1 N-terminal CTLH and K1 domains, but to a lesser extent on the ZMYND19 zinc finger (Fig. 5j).

### ZMYND19 associates with Raptor and RagA/C

**Figure 6:**
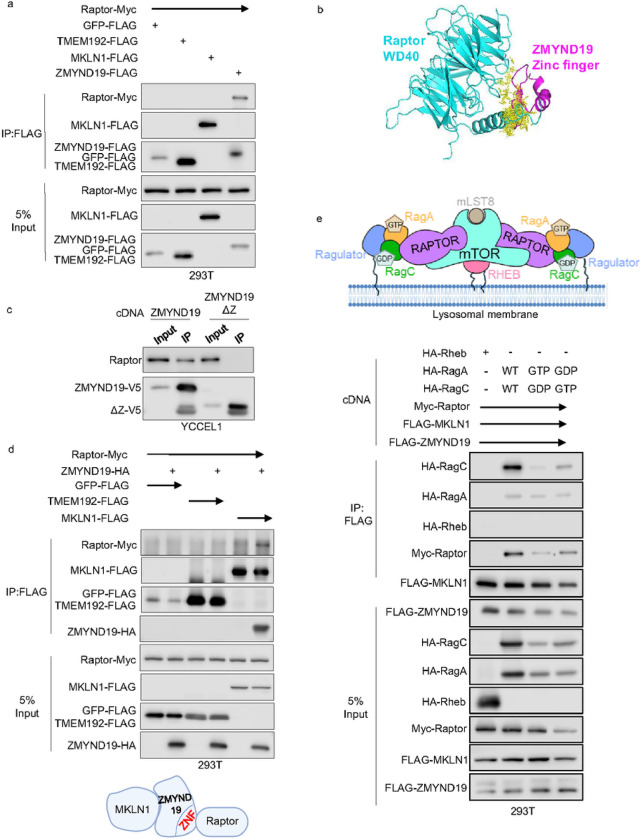
Interaction of ZMYND19/MKLN1 and Raptor. (A) Analysis of ZMYND19 and MKLN1 association with Raptor. Immunoblot analysis of 5% input vs anti-FLAG immunopurified complexes from HEK-293T transiently transfected with the indicated cDNAs for 24 hours. GFP and TMEM192 were used as negative controls for cytosolic and lysosomal membrane proteins, respectively. (B) Alphafold multimer 2 model of ZMYND19 zinc finger and Raptor WD40 association at 3.5 Å. The Raptor WD40 domain is shown in teal, the ZMYND19 zinc finger in magenta, and the predicted interaction interface is detailed in yellow. (C) Analysis of ZMYND19 zinc finger domain role in Raptor association. Immunoblots of 5% input versus anti-V5 immunopurified complexes from YCCEL1 that stably expressed the indicated V5-tagged ZMYND19 full length or zinc finger deletion mutant (ΔZ). (D) Analysis of ZMYND19 roles in association between MKLN1 and Raptor. Immunoblots of 5% input versus ant-FLAG immuno-purified complexes from 293T cells that transiently expressed the indicated epitope-tagged constructs. (E) Analysis of MKLN1/ZMYND19 association with RagA, RagC or RHEB. Top, schematic model of mTORC1 associated with Ragulator/RagA/C and RHEB complexes tethered to the lysosomal membrane, adapted from Rogala K., et al ^84^. GTP-bound RagA and GDP-bound RagC heterodimers, tethered to lysosomal membranes by regulator, associate with Raptor to recruit mTORC1 complexes, which can then be activated by membrane associated GTP-loaded RHEB. Below, immunoblot analysis of 5% input vs. anti-FLAG immunopurified complexes from 293T transiently transfected with the indicated FLAG-tagged ZMYND19 and MKLN1, Myc-tagged Raptor, HA-tagged Rheb and HA-tagged wildtype (WT) RagA and RagC or point mutants restricted to the GTP- or GDP-bound conformations^64^. Blots are representative of three independent replicates.

We hypothesized that ZMYND19 and/or MKLN1 interact with machinery that control mTOR activation at the outer lysosomal membrane. To test this, we performed co-immunoprecipitation analysis on lysates from cells that expressed FLAG-tagged GFP, TMEM192, MKLN1 or ZMYND19. Interestingly, FLAG-ZMYND19, but not any of the other baits, robustly co-immunoprecipitated Myc-tagged Raptor (Fig. 6a). In support, Alphafold Multimer 2 predicted that ZMYND19 associates with Raptor through its zinc finger domain (Fig. 6b **and Extended data Fig. S6a**). Consistent with this model, Raptor co-immunoprecipitated with full length, but to a significantly lesser extent, ΔZnF ZMYND19 (Fig. 6c **and Extended data Fig. S6b**). Furthermore, Raptor co-immunoprecipitated with MKLN1 only when it was co-expressed with ZMYND19 (Fig. 6d). Taken together, these data suggest that the ZMYND19 zinc finger supports association with MKLN1, Raptor and CTLH.

mTORC1 activity is tightly regulated by the Rag and Rheb^59–61^. Rag proteins are obligate heterodimers, in which RagA pairs with RagC and RagB pairs with RagD^62^. Rag heterodimers bind Raptor to recruit mTORC1 to lysosomes when amino acid supplies are abundant^63–65^ (Fig. 6e). We therefore tested whether ZYMND19 and MKLN1 associate with RagA/C or Rheb. To do so, FLAG-tagged MKLN1 and ZMYND19 were co-expressed with Myc-tagged Raptor and either HA-tagged Rheb or HA-RagA/C. Whereas Rheb did not appreciably associate with ZMYND19 and MKLN1, RagC and to a lesser extent RagA co-immunoprecipitated (Fig. 6e).

GTP-bound RagA/B and GDP-bound RagC/D serve to recruit mTORC1 to lysosome outer membrane sites, where mTORC1 is then activated by GTP-bound Rheb^63, 64, 66^ (Fig. 6e). To examine whether ZMYND19 and MKLN1 preferentially associate with either GTP loaded RagA/C state, we co-expressed RagA and RagC point mutants restricted to the GTP- or GDP-bound conformations^64^.Interestingly, while these expressed at lower levels than wildtype RagA or C, we nonetheless found that the RagC^GTP^ point mutant expressed with its RagA^GDP^ counterpart co-immunoprecipitated to a higher level with ZMYND19/MKLN1 than RagC^GDP^ co-expressed with RagA^GTP^ (Fig. 6e). These data suggest that ZMYND19 and MKLN1 may preferentially associate with the inactive Rag^GTP^ state. Since folliculin has GTPase activating activity towards RagC/D^67^, we also tested if it was important for mTORC1 inhibition by ZMYND19 and MKLN1. As expected, folliculin depletion strongly suppressed S6 phosphorylation, consistent with its roles in support of mTORC1 lysosomal recruitment. Interestingly, combined CRISPR folliculin and MAEA depletion further reduced S6 phosphorylation, suggesting that they may independently block mTORC1 (**Extended Data Fig. S6c**). However, MKLN1/ZMYND19 are unlikely to act through GATOR1, since MAEA KO inhibited mTORC1 in cells with combined KO of the NPRL2 GATOR1 catalytic subunit (**Extended Data Fig. S6d**).

### ZMYND19/MKLN1 block a late step of mTORC1 activation

**Figure 7:**
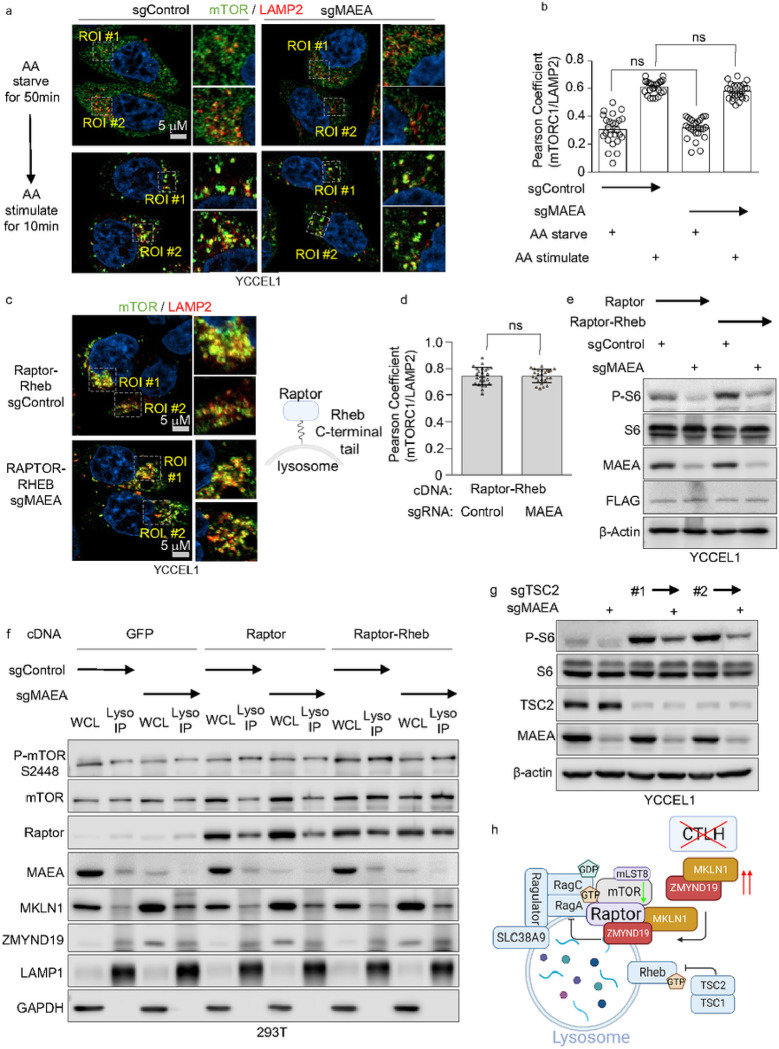
ZMYND19 and MKLN1 block a late stage in mTORC1 activation, distal to lysosomal membrane recruitment. (A) Analysis of MAEA depletion effects on amino acid stimulation driven mTOR lysosomal subcellular localization. Representative confocal microscopy images of YCCEL1 expressing control or MAEA sgRNAs and amino acid starved for 50 minutes and then cultured in amino acid free media (top) or with amino acid addback for 10 minutes (bottom). mTOR and lysosomal marker LAMP2 were stained with Alexa Fluor 488 (green) or Alexa Fluor 595 (red) conjugated antibodies, respectively. Zooms of two regions of interest (ROI) are shown to the right of each panel. (B) Analysis of mTORC1 and LAMP2 co-localization, using 25 cells as in (A). P values were calculated by Student’s t-test. Ns, non-significant. (C) Analysis of whether constitutively lysosomal membrane targeted Raptor can bypass mTORC1 blockade by MAEA depletion. Confocal microscopy of YCCEL1 expressing control or MAEA sgRNAs together with a Raptor fused to the Rheb C-terminal tail 15 amino acids, which serve as a lysosomal targeting sequence to constitutively drive Raptor to the lysosomes in an amino acid independent manner^68^ (as demonstrated in the model to the right). mTOR and lysosomal marker LAMP2 were stained with Alexa Fluor 488 (green) or Alexa Fluor 595 (red) conjugated antibodies, respectively. Zooms of two regions of interest (ROI) are shown to the right of each panel. (D) Analysis of mTORC1 and LAMP2 co-localization, using 25 cells as in (C). P values were calculated by Student’s t-test. (E) Analysis of Raptor-Rheb bypass of mTORC1 inhibition by MAEA depletion. Immunoblots of WCL from YCCEL1 that expressed Raptor or Raptor-Rheb cDNAs (as in C). (F) Analysis of Raptor-Rheb bypass effects on mTORC1 lysosomal recruitment and phosphorylation in control vs MAEA depleted cells. Immunoblot analysis of WCL for LysoIP obtained from 293T that expressed cDNAs encoding GFP, Raptor or Raptor-Rheb together with control or MAEA sgRNAs. (G) Analysis of whether TSC2 is necessary for mTORC1 inhibition upon MAEA depletion. Immunoblot analysis of WCL from YCCEL1 that expressed the indicated MAEA or TSC2 sgRNAs (#1 and #2). (H) Schematic model of CTLH, ZMYND19 and MKLN1 roles in mTORC1 regulation. A subpopulation of CTLH homes to lysosome regions. When MAEA is active, CTLH targets ZMYND19 and MKLN1 for proteasomal degradation. Upon loss of MAEA-dependent CTLH activity, ZMYND19 and MKLN1 associate at lysosome outer membrane sites, perhaps through ZMYND19 zinc-finger association with Raptor. Rather than blocking mTORC1 lysosomal recruitment, ZMYND19/MKLN1 block a late stage in mTORC1 activation. Blots are representative of three independent replicates.

We next asked whether MAEA knockout altered mTOR subcellular distribution under amino acid replete conditions. We therefore first asked whether MAEA KO blocked mTORC1 activation upon amino acid stimulation of cells starved of amino acids. Control or MAEA depleted YCCEL1 or 293T were grown in amino acid free media for 50 minutes, and then stimulated by amino acid add back for 10 minutes. Immunoblot analysis demonstrated that MAEA KO diminished the magnitude of S6 phosphorylation triggered by amino acid stimulation (**Extended data Fig. S7a**). Next, we performed confocal microscopy to define whether MAEA knockout altered mTOR subcellular localization following amino acid stimulation. Interestingly, mTOR and the lysosomal resident protein LAMP2 co-localized in both control and MAEA depleted cells following amino acid stimulation, suggesting that MKLN1/ZMYND19 do not block mTORC1 lysosomal recruitment (Fig. 7a–b).

To further test the model that ZMYND19/MKLN1 block mTORC1 activation at a step distal to lysosomal recruitment, we stably expressed either Raptor or a published Raptor-Rheb fusion protein (referred to hereafter as Raptor-Rheb), in which Raptor is fused to the Rheb C-terminal tail 15 amino acids. These Rheb residues serve as a lysosomal targeting sequence to dictate constitutive Raptor-Rheb lysosomal outer membrane localization, even upon amino acid starvation^68^. We validated that Raptor-Rheb, but not Raptor, drove mTOR lysosomal localization under amino acid starvation, as judged by co-localization with LAMP2 (**Extended data Fig. S7b**). Importantly, MAEA depletion did not perturb mTOR co-localization with LAMP2 (Fig. 7c–d, **Extended data Fig. S7c**), even though it did impair mTORC1 activity in cells that stably expressed Raptor-Rheb, as judged by S6 phosphorylation levels (Fig. 7e). Furthermore, MAEA depletion did not appreciably alter the amount of mTOR that co-purified with lysosomes in Lyso-IP analysis of cells with stable Raptor-Rheb expression (Fig. 7f).

The tuberous sclerosis complex, comprised of TSC1, TSC2 and TBC1D7 components, negatively regulates mTORC1 through Rheb GAP activity^40^. We therefore asked whether mTORC1 inhibition by ZMYND19 and MKLN1 was dependent on TSC2. However, CRISPR MAEA depletion inhibited S6 phosphorylation in YCCEL1 that were also depleted for TSC2 by either of two sgRNA approaches (Fig. 7g). We conclude that loss of CTLH activity blocks mTORC1 activation at a step distal to its lysosomal recruitment, but independently of TSC1/2 (Fig. 7h).

## Discussion

Despite intensive study, much remains to be learned about mechanisms that control mTORC1 activity. Here, we used a genome-wide CRISPR-Cas9 screen to identify targets whose depletion, together with alpelisib, blocked proliferation of an EBVaGC model with hyperactive PI3K/mTOR signaling. This analysis revealed that the multi-subunit E3 ligase CTLH is a major regulator of mTORC1 activity by suppressing levels of ZMYND19 and MKLN1. Upon loss of CTLH activity, ZMYND19 and MKLN1 were stabilized and associated with one another and with Raptor at lysosomal outer membrane sites. Since ZMYND19/MKLN1 did not perturb mTORC1 subcellular localization, but also did not depend on TSC2, our data suggest that they block a late stage of mTORC1 activation in a manner distinct from previously reported mechanisms.

MAEA was originally named Macrophage Erythroblast Attacher and found to have roles in erythrocyte development^69^. While our data agree with prior publications that MAEA is largely nuclear, we identified a cytoplasmic MAEA subpopulation that dynamically associated with lysosomes. In support, MKLN1 and ZMYND19 are mostly cytoplasmic, suggesting that they are regulated by a non-nuclear CTLH subpopulation. An interesting possibility is that a CTLH subpopulation may traffic to lysosomal regions in response to an environmental and likely nutritional cue. Notably, MKLN1 overexpression drives cytoplasmic trafficking of a CTLH subpopulation^37^. Thus, MKLN1 may direct CTLH to specific cytoplasmic targets^35^, which we now suggest include ZMYND19. We speculate that MKLN1 incorporation, perhaps together with additional post-translational modification, drives a CTLH subpopulation to lysosomal outer membrane sites. MKLN1 also has roles in actin- and microtubule-dependent GABA(A) receptor trafficking^70^, and MKLN1 may therefore play a roles in trafficking a subpopulation of CTLH to lysosomal outer membrane sites.

Little has remained known about ZMYND19. Our data suggest that ZMYND19 constitutively localizes to lysosomal outer membrane sites, where it recruits MKLN1 through its zinc finger domain. As ZMYND19 does not contain transmembrane domains and is not known to have lipid anchoring post-translational modifications, we speculate that ZMYND19 association with Raptor and RagC/D positions it, together with MKLN1, to block mTORC1 activity. We hypothesize that ZMYND19-bound MKLN1 is the substrate for CTLH in a manner dependent upon MAEA and potentially also RMND5a RING activity. In this manner, MKLN1 may serve as a CTLH substrate adaptor, but interestingly also as a CTLH substrate, together with ZMYND19. In support, CRISPR MKLN1 depletion significantly increased ZMYND19 abundance, whereas ZMYND19 depletion did not increase MKLN1 abundance. Since the ubiquitin E2 enzyme UBE2H nearly scored in our screen and is the human homolog of yeast GID3 that supports GID E3 ligase activity, our data suggest that MAEA RING-dependent ligase activity underlies the observed proteasomal turnover of MKLN1 and ZMYND19.

The CTLH RING subunits RMND5A and MAEA can each target MKLN1 for proteasomal degradation in HeLa cells^35^, where MKLN1’s half-life is ~24 hours, as opposed to ~8 hours in YCCEL1, perhaps suggesting a degree of cell-type specific regulation. Notably, RMND5A did not score in our CRISPR screen, but MAEA KO also depletes RMND5A in HeLa. Therefore, further studies will be required to determine whether RMND5A and MAEA have redundant activity towards MKLN1 and ZMYND19, or whether MAEA is uniquely able to target them. Notably, MKLN1 forms a tetramer, and four MKLN1 protomers can bind to two CTLH substrate receptor scaffolding modules *in vitro* to drive higher-order CTLH structures^71^. Whereas human CTLH assembles into complexes of 600–800 kDa, MKLN1 depletion shifts the complex towards 150–350 kDa^71^. We speculate that high molecular weight MKLN1-containing CTLH complexes target lysosome-bound ZMYND19 for degradation. Such a mechanism might account for our observation that CTLH transiently associates with lysosomes, since MKLN1 degradation at the lysosome may then alter CTLH structure and localization.

The CTLH homolog GID complex has major metabolic roles that enable yeast to rapidly adapt to the presence of glucose in the extracellular environment. When glucose is abundant, GID ubiquitinates and triggers proteasomal degradation of the gluconeogenic enzymes fructose 1,6-bisphosphatase (Fbp1), phosphoenolpyruvate carboxykinase (Pck1), cytoplasmic malate dehydrogenase (Mdh2) and isocitrate lyase (Icl1)^22^. Interestingly, GID can assemble with different substrate recognition factors. For instance, GID4 expression is de-repressed by the presence of extracellular glucose^72^, and GID4 then binds the catalytically inactive core complex to serve as a substrate receptor that drives polyubiquitination of specific gluconeogenesis enzymes^36, 47^. GID contains two RING finger domain containing proteins, GID2 and GID9, which form a heterodimer and whose human homologs are RMND5A and MAEA, respectively.

We speculate that human CTLH activity can be rapidly toggled in response to an as yet identified environmental signal. In this manner, when the nutrient is replete, CTLH degrades the MKLN1/ZMYND19 complex in order to license mTORC1 activation. However, when the nutrient is depleted, we anticipate that a post-translational modification disrupts this activity, enabling the ZMYND19/MKLN1 complex to accumulate, associate with Raptor and RagA/C to inhibit mTORC1. Such a mechanism would be distinct from GID regulation in yeast, in which glucose availability induces GID4 expression to drive E3 activity^36, 73^. Thus far, we have not observed changes in CTLH activity towards ZYMND19/MKLN1 upon glucose withdrawal, suggesting that CTLH may not function in a glucose sensing pathway. Intriguingly, carbon stress regulates assembly of an anticipatory form of the GID E3 ligase, which then awaits expression of the substrate receptor for activation of its activity^47^. Thus, it will be of interest to test whether CTLH assembly might instead be negatively regulated by carbon stress. Alternatively, CTLH phosphorylation by casein kinase 2 regulates UBE2H/CTLH pairing^74^, and this may enable cross-talk between receptor driven kinase signaling, CTLH activity and mTORC1.

How do ZMYND19 and MKLN1 block mTORC1? Our data suggest that they act at a late step in mTORC1 activation, after it has been recruited to lysosomes. This suggests that CTLH activity towards ZMYND19 and MKLN1 is unlikely to be regulated by known signals that alter mTORC1 lysosomal recruitment, including amino acid sensing pathways that signal through CASTOR1, Sestrin1/2 or SAMTOR. In support, CRISPR KO of the GATOR1 catalytic subunit NPLR2 did not preclude mTORC1 inhibition upon subsequent MAEA KO. Given our observation that ZMYND19 associates with Raptor and with GDP-loaded RagC, the ZMYND19/MKLN1 complex is well positioned to block either Rheb GTP loading or Rheb-mediated allosteric mTORC1 activation. However, mTORC1 was inhibited by MAEA KO in cells lacking TSC2, suggesting that ZYMND19/MKLN1 does not act by increasing TSC1/2 GAP activity towards Rheb. ZMYND19/MKLN1 may instead prevent Raptor binding to the Tor signaling sequence (TOS) motif found in the 4EBP1 and S6K1 substrates and also in mTORC1 regulators^75,76^. Alternatively, ZMYND19/MKLN1 may preclude association between Ragulator-bound mTORC1 and Rheb. Since we did not detect association between ZMYND19/MKLN1 and Rheb, such a mechanism would likely operate by preventing association of ZMYND19/MKLN1-bound mTORC1 complexes with Rheb. ZMYDN19/MKLN1 may instead prevent obligatory Rheb-driven allosteric activation of mTORC1 kinase activity or mTORC1 substrate-recruitment, in a manner similar to PRAS40 in the absence of insulin signaling^77^.

Our results highlight an interesting difference upon MAEA depletion in human cells versus RMND5A depletion in the *C. elegans* GID complex. KO of the other GID RING ligase subunit RMND5A increased AMP kinase expression and activation, which reduced mTOR activity, increased autophagic flux and increased *C. elegans* lifespan^49^. While AMP kinase was identified as a *C. elegans* GID substrate, we did not detect increased AMPK kinase levels by our proteomic or immunoblot analyses of MAEA KO vs control human cells. Thus, it is possible that this GID role has not been conserved in human cells, or may instead require loss of both MAEA and RMND5A ubiquitin ligase activity. Similarly, CTLH can target the transcription repressor HBP1 for degradation to support cell cycle^54^, though we did not observe significantly increased HBP1 levels in YCCEL1 upon MAEA KO, perhaps indicative of a cell-type specific HBP1 regulatory role.

CTLH perturbation downregulated mTORC1 in two EBVaGC models and also in several EBV-negative cell lines. Since CTLH is expressed in a wide range of human cell types and tissues, we suspect that it supports mTORC1 in diverse human cellular contexts, particularly with hyperactive PI3K signaling. This may relate to the observation that CLTH subunits are overexpressed in a variety of human tumors, and that elevated WDR26 levels correlate with poor prognosis^78^. Our results therefore suggest CTLH may be a therapeutic target. For instance, GID4 small molecules binders were recently identified^79^, and it is likely that CTLH inhibitors can be developed. While germline MAEA KO causes myeloproliferative disease in mice^80^, it is possible that CTLH inhibition in combination with low-dose FDA-approved PI3K inhibitors such alpelisib may not have this effect. Thus, CTLH inhibition together with PI3K blockade warrants further investigation as a potential therapeutic modality for EBVaGC and for other tumors dependent upon hyperactive PI3K/mTOR signaling.

In summary, a human genome-wide CRISPR/Cas9 screen identified that KO of multiple CTLH E3 ligase subunits blocked EBVaGC proliferation, together with alpelisib. We identified that CTLH substrates ZMYND19 and MKLN1 formed a complex that associated with Raptor and RagA/C. In the absence of CTLH activity, ZMYND19 and MKLN1 were stabilized and inhibited mTORC1 at a step distal to its lysosomal membrane recruitment. These studies identify a novel EBVaGC therapeutic target whose inhibition may also be synergistic with PI3K blockade in tumors with aberrantly elevated PI3K activity, and identify a pathway by which CTLH-dependent proteasomal activity tunes mTORC1 activity at the lysosomal membrane.

## Methods

### Cell culture

The EBV+ gastric cancer cell line YCCEL1 was obtained from Elliott Kieff. The EBV+ cell line SNU719 was obtained from Adam Bass. The EBV− gastric cancer cell line HGC-27 was obtained from Sigma, SNU-1 and SNU-16 were from ATCC. HEK-293T was obtained from ATCC. Cell lines with stable Streptococcus pyogenes Cas9 expression were generated by lentiviral transduction and blasticidin selection (5 mg/mL), as previously described ^85^. Cells were cultured in a humidified incubator with 5% CO_2_ at 37°C. The cells were routinely tested and certified as mycoplasma-free using the MycoAlert kit (Lonza).

YCCEL1 cells were grown in Eagle’s Minimum Essential Medium (EMEM) medium (ATCC) with 10% fetal bovine serum (FBS, Gibco). SNU719 and SNU16 cells were grown in RPMI 1640 medium (Gibco, Life Technologies) with 10% FBS. 293T was grown in Dulbecco’s Modified Eagle’s Medium (DMEM) with 10% FBS. HGC-27 were grown in EMEM (ATCC) with 2mM Glutamine (Gibco, Life Technologies), 1% Non- Essential Amino Acids (NEAA, Gibco, Life Technologies), and 10% FBS. BYL719 (Alpelisib, Cat#16986, Cayman chemical) was used at 0.5mM. Antibodies used in the study are listed in the Reporting Summary.

### HEK-293T transfection

HEK-293T cells were transfected as follows. 2 million cells were seeded in 10 cm dishes (Corning). After 24h, pRK5 based plasmids were transfected with the Effectene transfection reagent (Qiagen, 301425) according to manufacturer’s protocol. Empty pRK vector was used to normalize the level of DNA to 2ug across transfection conditions. Cells were collected 24–30h post-transfection. The following amounts of cDNA were used in the indicated figures. Fig.6A, 500ng Raptor-MYC, 50ng GFP-FLAG, 50ng TMEM192-FLAG, 300ng ZMYND19-FLAG, or 300ng MKLN1-FLAG. Fig. 6C, 500ng Raptor-MYC, 50ng GFP-FLAG, 50ng TMEM192-FLAG, 300ng ZMYND19-FLAG or 300ng MKLN1-FLAG. Fig.S5C 300ng ZMYND19-HA, 50 ng GFP-FLAG, 50 ng TMEM192-FLAG, 300 ng MKLN1-FLAG or 300 MKLN1-FLAG truncation mutants. Fig.S5D 300ng ZMYND19-HA, 50 ng GFP-FLAG, 50 ng TMEM192-FLAG, 300 ng MKLN1-FLAG or 300 MKLN1 truncates with FLAG tag. Fig.S6C 500ng Raptor-myc, 300ng ZMYND19-FLAG, or 300 ng MKLN1-FLAG, 100ng Rheb-HA, 100ng RagA or 100ng RagC.

#### Chemicals.

Alpelisib (BYL719) was purchased from Cayman chemical (Cat#16986) and was used at 0.5mM unless otherwise indicated. ML-210 was purchased from Cayman Chemicals (Cat#23282) and used at 5mM. Ferrostatin-1 (Fer-1) was purchased from Sigma-Aldrich (Cat#SML0583) and used at 10mM. Propidium iodide was purchased from Invitrogen (Cat#P03566) and used at 5μg/ml in cell cycle analysis, 2-NBDG from ThermoFisher (Cat#N13195) and used at 10 mg/mL in glucose uptake assays, puromycin from Thermofisher (CatA1113803) was used at 10 μg/mL in puromycin chase assays, and cycloheximide from R&D systems (Cat#0970/100) was used at 50μg/mL for protein half-life and as a control for puromycin chase analysis. Doxycycline was used at 250 ng/ml.

### Lentivirus production and transduction

0.3 million HEK-293T cells were seeded in the 6-well plates (Corning). After 24h, cells were co-transfected with 500 ng of lentiviral plasmids (400 ng of psPAX2 and 150 ng of VSV-G) using TransIT-LT1 Transfection Reagent (Mirus Bio). At 48 and 72 hours post-transfection, supernatants containing lentivirus were filtered through a 0.45 μm SFCA syringe filter (CellTreat) and transferred to target cells, together with fresh medium. Transduced cells were selected for 5 days with puromycin (1:3000, 3mg/ml, Gibco) or 10 days with hygromycin (1:500, 100mg/ml, Gibco).

### Cell lysis and immunoprecipitation

For anti-V5-IP, cells were transduced with lentivirus produced in 293 cells, using pLX-TRC313-MAEA, pLX-TRC313-MKLN1, pLX-TRC313-ZMYND19, or pLX-TRC313-GFP, as described in the lentivirus production section. 10 million cells were rinsed with PBS and lysed by ice cold IP lysis buffer (ThermoFisher, #87788) with EDTA-free protease inhibitor (Roche), and cleared by 14,000 rpm centrifugation for 10 min at 4°C in a benchtop microcentrifuge. Lysates were incubated with 30 μL of the pre-cleared beads per 1mL of lysate for 3h at 4°C. Supernatants were then subjected to immunoprecipitation. For V5 pulldowns, cell lysates were incubated with anti-V5-tag magnetic beads (MBL) (30 mL beads/1ml samples) for 3h at 4°C. For anti-Flag-IP, anti-Flag M2 antibody (Sigma, F1804) was incubated with 25 ml Pierce protein A/G magnetic beads per 1 ml of lysate (ThermoFisher, 88803) for 1h at room temperature, washed with 1xTBST once and 1xTBST with 0.5mM NaCl 3 times. For anti-HA IP, Pierce magnetic anti-HA beads (ThermoFisher, 88837) were washed three times with lysis buffer, and 1 ml lysate was incubated with 30 μL of the beads for 3h at 4°C. Beads were then washed three times, once with IP lysis buffer (ThermoFisher), twice with lysis buffer 30ml of beads in 1ml lysate for each sample with 0.5 mM NaCl. Immunoprecipitated proteins were boiled for 5 minutes in Laemmli Sample Buffer (Biorad) and subject to SDS/PAGE and immunoblot analysis.

### Growth Curve analysis

For growth curve analysis, cells were counted and then normalized to the same starting concentration. Live cell numbers were quantitated at each timepoint by cell counting with the TC20 automatic cell counter (Bio-Rad). Fold change of live cell number at each timepoint was calculated as a ratio of the value divided by the input value.

### CRISPR/Cas9 screen

The Broad Institute Brunello sgRNA lentivirus library was used to generate YCCEL1 libraries. Briefly, 130 million YCCEL1 stably expressing Cas9 were transduced with the Brunello library at a multiplicity of infection of 0.3 by spinoculation in 12-well plates at 300g for 2 hours in the presence of 4 μg/μl of polybrene. The library contains four distinct sgRNAs of each human gene and multiple control sgRNAs. Cells were incubated at 37°C with 5% CO_2_ for 6 hours, at which point the EMEM media was exchanged to remove polybrene. 48 hours later, puromycin was added to select transduced cells at 3 mg/mL. After 5 days, cells were then cultured in the presence of 0.5mM alpelisib versus DMSO vehicle control. Cells were passaged every 3 days in fresh alpelisib or DMSO for 14 days. Genomic DNA was then harvested with the Blood and Cell Culture DNA Maxi Kit (Qiagen) from 40 million cells per screen replicate, according to the manufacturer’s protocol. PCR amplified sgRNA abundances were quantified by an Illumina Hisses sequencer^26^ at the Broad Institute. The STARS algorithm was applied to calculate hit statistical significance, using a stringent cutoff of q < 0.05 (p-value adjusted for the False Discovery Rate).^26^

### Screen validation

sgRNA oligos were obtained from Integrated DNA Technologies and cloned into the pLentiGuide-Puro vector (Addgene plasmid #52963, a gift from Feng Zhang). Lentiviruses were produced in 293T cells by co-transfection of pLentiGuide-puro with psPAX2 and VSV-G. Transduction and puromycin selection were done as described as above. Live cell numbers were quantitated using propidium-iodide stating and measurement by a TC20 automatic cell counter (Bio-Rad). sgRNA sequences used are listed in Supplementary Information.

### Flow Cytometry

FACS analysis was performed on a BD FACSCalibur instrument and analyzed by FlowJo V10. For cell cycle analysis, cells were fixed in 70% ethanol overnight at 4^●^C, washed twice with PBS, treated with staining buffer (propidium iodide 5μg/ml, RNase A 40μg/ml and 0.1% Triton X-100 in PBS) for 30 minutes at room temperature. For glucose uptake analysis, live trypsinized cells were incubated with 10 mg/mL 2-NBDG in complete media at 37^●^C and tested every 15 minutes for 2h.

### Immunoblot analysis.

Immunoblot was performed as previously described.^85^ In brief, whole cell lysates were prepared with ice cold RIPA lysis buffer (NaCl, 150mM; NP40, 1%; DOC, 0.5%; SDS, 0.1%; Tris (pH7.4), 50mM) microcentrifuged on top speed for 5 minutes at 4^●^C, and boiled with Laemmli Sample Buffer (Biorad). Samples were separated by SDS-PAGE electrophoresis, transferred onto the nitrocellulose membranes, and blocked with 5% milk or 5% BSA in TBST buffer. Membranes were washed three times with TBST and then probed with primary antibodies at 4^●^C overnight. Membranes were then washed three times with TBST and incubated with secondary antibody for 1 h at room temperature. Membranes were washed three times with TBST and then developed by incubation with ECL chemiluminescence for 30s (Millipore). Images were captured by a Licor Fc platform. Band intensities were measured with Image Studio Lite Version 5.2. All antibodies used in this study were listed in the key resources table. The following antibodies were used in this study. Phospho-Akt (Ser473) (D9E, Cat#4060S), Phospho-Akt (Thr308) (244F9, Cat#4056S), Akt (Cat#9272S), GPX4 (Cat#52455S), anti-Phospho-p70 S6 Kinase (Thr389) (108D2, Cat#9234S), p70 S6 Kinase (Cat#9202S), Phospho-4E-BP1 (Thr37/46) (236B4, Cat#2855S), 4E-BP1 (53H11, Cat#9644S), V5-Tag (D3H8Q, Cat#13202S), Phospho-AMPKα (Thr172) (40H9, Cat#2535S), AMPKα (D5A2, Cat#2532S), Phospho-eIF2α (Ser51, Cat#9721S), eIF2α (D7D3, Cat#5324S), Raptor (24C12, Cat#2280S), HA-Tag (C29F4, Cat#3724S), GAPDH (D16H11, Cat#5174S), Myc-Tag (9B11, Cat#2276S), Myc-Tag (71D10, Cat#2278S), DYKDDDDK Tag (Cat#2368S), NPRL2 (D8K3X, Cat#37344S), FLCN (D14G9, Cat#3697S), Phospho-mTOR (Cat#2971S), -mTOR (7C10, Cat#2983S), anti-Rabbit IgG HRP-coupled secondary antibody (Cat#7074S), anti-Mouse IgG HRP-coupled secondary antibody (Cat#7076S), anti-Rat IgG HRP-coupled secondary antibody (Cat#7077S) were purchased from Cell Signaling Technology. KRAS (Cat# 12063-1-AP) and USP7 (Cat# 66514-1-Ig) antibodies were purchased from Proteintech; Beta-actin (Cat#664802) and ATF4 (Cat#693901) antibodies were purchased from Biolegend. MAEA (Cat#AF7288) antibody was purchased from R&D Systems. Anti-Sheep IgG HRP-coupled secondary antibody (Cat#A3415), anti-puromycin antibody (12D10, Cat#MABE343) and anti-GFP (GF28R, Cat# MA5-15256) antibody were purchased from Millipore Sigma. Anti-MKLN1 (C-12, Cat#sc-398956), anti-ZMYND19 (E-4, Cat#sc-398514) and anti-LAMP1 (H4A3, Cat#sc-20011) antibodies were purchased from Santa Cruz Biotechnology. Anti-LAMP-2 antibody (Cat#H4B4) was purchased from DSHB. Alexa Fluor^®^ 594 AffiniPure Donkey Anti-Mouse IgG (H+L) (Cat#715-585-150) and Alexa Fluor^®^ 488 AffiniPure Goat Anti-Rabbit IgG (H+L) (Cat#111-545-144) were purchased from Jackson ImmunoResearch.

### RNAseq analysis

Total RNA was isolated by the RNeasy Mini kit (Qiagen), following the manufacturer’s protocol. Removal of the residual genomic DNA contamination was included in the RNA preparation steps. To construct indexed libraries, 1 mg of total RNA was used to select polyA mRNA using the NEBNext Poly(A) mRNA Magnetic Isolation Module (New England Biolabs), followed by library construction via the NEBNext Ultra RNA Library Prep Kit (New England Biolabs). Three replicates were used for each condition. Libraries were multi-indexed, pooled and sequenced on an Illumina NextSeq 500 sequencer, using single-end 75 bp reads (Illumina) at the Dana-Farber Molecular Biology core. Adaptor-trimmed Illumina reads for each individual library were mapped to the human GRCh37.83 transcriptome using STAR2.5.2b^86^. Feature Count was used to estimate the number of reads mapped to each contig^87^. Only transcripts with at least 5 cumulative mapping counts were used in this analysis. DESeq2 was used to evaluate differential expression (DE)^88^. Each DE analysis used pairwise comparison between the experimental and control groups. Differentially expressed genes were identified and a p value <0.05 cutoff was used. Differentially expressed genes were subjected to Enrichr analysis^89^. Top Enrichr terms and volcano plots were visualized using Graphpad Prism 7.

### MAEA cDNA rescue

MAEA sgRNA PAM site mutations were introduced into the entry vector pENTR223-MAEA (DNASU#HsCD00510985), containing the DNA sequence encoding N-terminally V5-tagged full-length human MAEA. Point mutagenesis was performed using the Q5 site directed mutagenesis kit (New England Biolabs), designated as pENTR223-MAEA-PAM. Genomic sequences with PAM mutations are underlined, CCAGGAGTACCCGACCCTCAAAG and GCGTTGCGGCTACTACAACACAG. MAEA cDNAs were transferred to the lentiviral destination vector pLX-TRC313 (Broad Institute) using Gateway LR Clonase II Enzyme Mix (Thermo Fisher Scientific). This appended the sequence for a V5-tag onto the MAEA C-terminus. The lentivirus vector pLXTRC313-GFP, which stably expresses a GFP cDNA, was used as a control. YCCEL1 Cas9 cells were transduced with either pLX-TRC313 GFP or MAEA encoding lentiviruses. Cells were selected with hygromycin (100mg/ml, Gibco) for 2 weeks. Heterogenous protein expression was confirmed by immunoblot using anti-V5 tag antibody. CRISPR targeting of endogenous MAEA was then performed as described above. Subsequently, cells were selected with puromycin (1:3000, 3mg/ml, Gibco). sgRNA sequences are listed in the Supplementary Information.

### Targeted Metabolite profiling

3×10^6^ of YCCEL1 expressing control or MAEA sgRNA were seeded into a T75 flask with 15 mL of EMEM with 10% FBS. Two days after seeding, cells were treated with 0.5mM alpelisib or DMSO for 30h. Cells were collected with scrapers (Corning#353089) and washed with 5 mL of room temperature PBS. Then, pellets were resuspended in 1 mL of dry ice cold 80% methanol, incubated at −80°C for 30 min and centrifuged at 21,000 × g for 5 minutes at 4°C. Supernatants were collected in pre-chilled tubes and stored at −80°C. 6 replicates were included for each treatment. On the day of analysis, supernatants were incubated on ice for 20 min, clarified by centrifugation at 21,000 × g at 4°C, and dried down with a speed vacuum concentrator (Savant SPD 1010, Thermofisher Scientific). Samples were re-suspended in 100μL of 60/40 acetonitrile/water, vortexed, sonicated in ice-cold water, and incubated on ice for 20 min. Following centrifugation at 21,000×g for 20 min at 4°C, supernatants were collected for pooled QC. Metabolite profiling was performed at the Beth Israel Deaconess Mass Spectrometry Core. Specifically, samples were re-suspended using 20 uL HPLC grade water for mass spectrometry. 5–7 μL were injected and analyzed using a hybrid 6500 QTRAP triple quadrupole mass spectrometer (AB/SCIEX) coupled to a Prominence UFLC HPLC system (Shimadzu) via selected reaction monitoring (SRM) of a total of 300 endogenous water-soluble metabolites for steady-state analyses of samples. Some metabolites were targeted in both positive and negative ion mode for a total of 311 SRM transitions using positive/negative ion polarity switching. ESI voltage was +4950V in positive ion mode and −4500V in negative ion mode. The dwell time was 3 ms per SRM transition and the total cycle time was 1.55 seconds. Approximately 9–12 data points were acquired per detected metabolite. For targeted 13C flux analyses, isotopomers from ~140 polar molecules were targeted with a total of 460 SRM transitions. Samples were delivered to the mass spectrometer via hydrophilic interaction chromatography (HILIC) using a 4.6 mm i.d × 10 cm Amide XBridge column (Waters) at 400 μL/min. Gradients were run starting from 85% buffer B (HPLC grade acetonitrile) to 42% B from 0–5 minutes; 42% B to 0% B from 5–16minutes; 0% B was held from 16–24 minutes; 0% B to 85% B from 24–25 minutes; 85% B was held for 7 minutes to re-equilibrate the column. Buffer A was comprised of 20 mM ammonium hydroxide/20 mM ammonium acetate (pH=9.0) in 95:5 water:acetonitrile. Peak areas from the total ion current for each metabolite SRM transition were integrated using MultiQuant v3.2 software (AB/SCIEX). Metabolites with CV<30% in pooled QC were used for the statistical analysis. The quality of integration for each metabolite peak was reviewed. Metabolites with p-values < 0.05, log2(fold change)>1 or <-1 were used for pathway analysis using MetaboAnalyst 5.0 (https://www.metaboanalyst.ca/ MetaboAnalyst/ModuleView.xhtml). Heatmaps of metabolites in the pathways were generated by feeding Z-score values into Morpheus software (https://software.broadinstitute.org/morpheus/).

### Seahorse Analysis

YCCEL1 were seeded in 96-well plates at 50,000 per well. After 24h, each group was treated with DMSO or 0.5mM alpelisib for 8h. The sensor cartridge plate was hydrated overnight with XF calibrant. 1.5 μM oligomycin, 0.5 μM FCCP and 0.5 μM antimycin were added at the indicated timepoints. Oxygen consumption rates (OCR) and Extracellular acidification rate (ECAR) were measured at each time point by Agilent seahorse XF96 analyzer using a Seahorse XF96 sensor cartridge. The Seahorse XF Cell Mito Stress Test Kit was purchased from Agilent (Cat#103015–100). Oligomycin, CCCP and antimycin were used at 1.5 μM, 0.5 μM and 0.5 μM respectively for Seahorse analysis.

### Proteomic analysis

Cell pellets were lysed in 400 μl of lysis buffer containing 50 mM Tris pH 7.5, 1% (w/v) SDS, 150 mM NaCl, 1 mM EDTA supplemented with EDTA-free protease inhibitor (Roche). The lysate was pulse sonicated and centrifuged at 15,000 g for 15 min at 4°C. Samples were quantified using the Pierce BCA protein assay kit (ThermoFisher Scientific, #23225). 30 μg of each sample was reduced with tris(2-carboxyethyl) phosphine, alkylated with iodoacetamide, and digested with trypsin. The digests were desalted using BioPureSPN C18 spin column (The Nest Group) and labeled with TMT 10plex isobaric label reagents (ThermoFisher Scientific, #90110) following the manufacturer’s instructions. After labeling, an equal amount peptide from each sample was pooled together, fractionated into 13 fractions using Pierce high pH reverse-phase peptide fractionation kit (ThermoFisher Scientific, # 84868) and dried in a SpeedVac concentrator. After resuspended in 1% formic acid, samples were analyzed on a Q Exactive HF mass spectrometer (Thermo Scientific) coupled to an UltiMate 3000 RSLCnano HPLC system (Thermo Scientific). Briefly, samples were injected into a PepMap100 trap column (0.3 × 5 mm packed with 5 μm C18 resin; Thermo Scientific), and peptides were separated by reversed phase HPLC on a BEH C18 nanocapillary analytical column (75 μm i.d. × 25 cm, 1.7 μm particle size; Waters) using a 2.5-h gradient formed by solvent A (0.1% formic acid in water) and solvent B (0.1% formic acid in acetonitrile). Eluted peptides were analyzed by the mass spectrometer in data-dependent mode with survey scan from 350 to 1800 m/z followed by MS/MS scans at 45,000 resolution on the 15 most abundant ions. The automatic gain control (AGC) targets for MS1 and MS2 were set at 3E6 and 2E5 ions, respectively. The maximum ion injection times for MS1 and MS2 were set at 50 and 120 ms, respectively. The isolation window was 0.7 m/z, normalized collision energy was 30%, first mass was fixed at 110 m/z, and charge-state screening was used to reject unassigned, single and >7 charged ions. Proteins were identified and quantified using Proteome Discoverer v2.4 (Thermo Scientific). Finally, MS/MS spectra were searched against the SwissProt human and EBV protein databases (June 2021) and consensus identification lists were generated with false discovery rates set at 1%.

### Protein half-life analysis

Cycloheximide chase analysis was performed as follows. YCCEL1 were seeded in 6-well plates at a density of 300,000 cells/ml. 48h later, cells were treated with 50μg/mL cycloheximide vs DMSO control. Cells were then harvested at the appropriate timepoints, lysed, and subject to immunoblot analysis.

### Puromycin chase analysis

YCCEL1 were seeded into 6-well plates at a density of 300,000 cells/ml. 48h later, cells were treated with 10 μg/mL puromycin for 20 minutes at 37°C. WCLs were prepared and analyzed by immunoblot, using an anti-puromycin monoclonal antibody to visualize polypeptides newly synthesized during the puromycin pulse.

### In vitro transcription

The IVT DNA fragments were synthesized from IDT, with a T7 promoter (TAATACGACTC ACTATAGG) at 5’ end followed by the coding sequence of interest followed by the sequence encoding the V5 tag (GGTAAGCCTATCCCTAACCCTCTCCTCGGTCTCGAT TCTACG), followed by a 3’-UTR (GCTCGCTTTCTTGCTGTCCAATTTCTATTAAAGGTTCCTTTGTTCCCTAAGTCCAACTACTAAACTGGGGGATATTATGAAGGGCCTTGAGCATCTGGATTCTGCCTAATAAAAAACATTTATTTTCATTGC) and polyA sequence (AAAAA). To add multiple polyA tails, fragments were amplified by the IVT-R reverse primer and forward primers to the T7 promoter and 5’ sequence of the gene of interest (the sequences for the IVT-MKLN1-F, IVT-ZMYND19-F and IVT-GFP-F primers are listed in the supplement). The template was subject to *in vitro* transcription, using the mMACHINE^™^ T7 Transcription kit (Thermofisher, AM1344), as described previously^90^. Briefly, DNA fragments were amplified and the correct length was confirmed by gel electrophoresis. Following clarification by Microspin G-25 columns (Cytiva, #27532501), Products were further cleaned by phenol: chloroform: isoamyl alcohol (25:24:1) extraction. The purified DNA was then precipitated with 0.3M sodium acetate (pH5.2, Sigma) and 5ug/ml glycogen (Invitrogen) in 75% ethanol, washed with 70% ethanol, air dried, and dissolved with nuclease-free water. DNA was then used for *in vitro* transcription reactions, according to manuscript’s protocol using 10ul 2xNTP/CAP, 0.75ml GTP, 2ml 10xreaction buffer, 1mg DNA template and 2ml enzyme mix at 37 °C for 3 hours. Reactions were stopped by addition of 2ml DNase and incubation at 37°C for 15 minutes. Finally, mRNAs were purified by phenol:chloroform extraction and isopropanol precipitation.

### mRNA Electroporation

The Neon Transfection System (Thermo Fisher) was used to deliver in vitro transcribed mRNA. Based on gene length, 500ng GFP, 500ng ZMYND19 or 1500ng MKLN1 encoding mRNA was mixed with 0.6 million cells in 10ul Buffer T (Thermo Fisher) per reaction. The electroporation parameters were 1350v pulse voltage, 20 ms of pulse width and 2 pulses for YCCEL1; 1500v, 30 ms and 1 pulse for HEK-293T. Cells were then rapidly returned to the incubator and cultured as described above.

For delivery of RNAs for CRISPR editing, pre-designed crRNA and tracrRNA were ordered from IDT. 0.6ml of crRNA-tracrRNA (0.12 nmol of crRNA and 0.12 nmol tracrRNA for each reaction) was mixed with 0.6 million cells in 10uL Buffer T. Cells were subjected to electroporation using the following Neon program (1350v pulse voltage, 20 ms pulse width, 2 pulses for YCCEL1, and 1500v, 30 ms and 1 pulse for HEK-293T) and a 10 mL tip. crRNA sequences are listed in Supplementary Information.

### Live cell imaging

YCCEL1 were plated at a density of 300,000 cells/ml in glass bottom 35 mm dishes (MATTEK#P35GC). After 48h, media was exchanged and cells were transfected with cDNA encoding an MAEA-GFP chimera (Sino Biological) using TransIT (Mirusbio#MIR2306), according to manufacturer’s protocol. After 24 hours, cells were stained with Lysotracker 0.5mM (Invitrogen#L7528) at 37°C for 30min. Then, image acquisition was performed, using a Zeiss LSM 800 microscope with parameters LSM scan speed 8 and frame time 1.86s. Zeiss Zen Lite (Blue) software was used for image analysis.

### lmmunofluorescence

Seeded cells were permeabilized with 0.5% Triton X-100/PBS for 5 mins, blocked with 1% BSA/PBS for 1h at room temperature and incubated with anti-mTOR (1:250) and anti-LAMP2 (1:150) primary antibodies for 1h at 37°C. Then, cells were washed three times with TBS and incubated with anti-mouse (1:250) and anti-rabbit (1:250) secondary antibodies for 1h at 37°C in the dark. Cells were washed three times with TBS and stained with DAPI (1:5000) for 10min. Image acquisition was performed by a Zeiss LSM 800 instrument. Image analysis was performed with Zeiss Zen Lite (Blue) software. Image J Coloc2 was used to score the colocalization of mTOR and lysosomes.

### Lysosomal immunopurification

LysoIP was performed as described previously^57^. Briefly, 35 million YCCEL1 stably expressing HA-tagged TMEM192 were harvested by scrapers on ice, washed twice with ice cold TBS, and resuspended in 1 ml of ice cold KPBS (136mM KCl, 10mM KH2PO4, pH 7.25). Cell suspensions were centrifuged at 1000×g for 2 mins at 4°C. Pelleted cells were resuspended in 950mL of ice cold KPBS, and 25uL was reserved for a whole cell fraction. The remaining cells were homogenized on ice with a 2 ml homogenizer. The homogenate was centrifuged at 1,000g for 2min at 4°C. Supernatants were incubated with 100 ml of anti-HA magnetic beads (Pierce/Thermo) for 10 mins at 4°C with rotation. Beads were washed with ice cold KPBS three times and then eluted using 1X SDS loading buffer for immunoblot analysis.

### Structural prediction

Protein structural prediction for ZMYND19, MKLN1 or Raptor was performed with AlphaFold Collab-Multimer.^91^ Structure visualization was performed with Polo version 2.5.

### Amino acids starvation and stimulation

YCCEL1 cells were washed with PBS and then starved by incubation in amino acid-free EMEM with 10% dialyzed FBS (Gibco) at 37°C for 50 minutes. Amino acid-free EMEM was prepared based on formulation of ATCC 30–2003. Amino acid stimulation was then performed by adding 100X MEM amino acid solution (1:100, ThermoFisher) at 37°C for 10min.

### Transmission electron microscopy

A pellet of cells was fixed for at least 2 hours at room temperature in fixative (2.5% Glutaraldehyde 1.25% Paraformaldehyde and 0.03% picric acid in 0.1 M sodium cacodylate buffer (pH 7.4)), washed in 0.1 M cacodylate buffer and post-fixed with 1% Osmium tetroxide (OsO4)/1.5% Potassiumferrocyanide (KFeCN6) for 1 h, washed 2x in water, 1x Maleate buffer (MB) 1x and incubated in 1% uranyl acetate in MB for 1 h followed by 2 washes in water and subsequent dehydration in grades of alcohol (10 mins each; 50%, 70%, 90%, 2×10 mins 100%). Samples were then put in propylene oxide for 1 h and infiltrated overnight in a 1:1 mixture of propylene oxide and TAAB (TAAB Laboratories Equipment Ltd, https://taab.co.uk). The following day the samples were embedded in TAAB Peon and polymerized at 60°C for 48 h. Ultrathin sections (about 60 nm) were cut on a Reichert Ultracet-S microtome, picked up on to copper grids stained with lead citrate and examined in a JEOL 1200EX Transmission electron microscope or a TecnaiG2 Spirit Biotin and images were recorded with an AMT 2k CCD camera.

### Data Visualization

Figures were drawn with GraphPad, Bio render, Powerpoint and ggplot2 in R.^92^

## Data Availability

All plasmids generated for this study will be made available on request where permissible. CRISPR screen data will be shared upon request.
